# GAS6‐expressing and self‐sustaining cancer cells in 3D spheroids activate the PDK‐RSK‐mTOR pathway for survival and drug resistance

**DOI:** 10.1002/1878-0261.12109

**Published:** 2017-07-26

**Authors:** Christine Baumann, Axel Ullrich, Robert Torka

**Affiliations:** ^1^ Department of Molecular Biology Max‐Planck‐Institute of Biochemistry Martinsried Germany; ^2^ Institute of Physiological Chemistry University Halle‐Wittenberg Halle (Saale) Germany

**Keywords:** 3D spheroid, AXL, drug resistance, GAS6, RTK, TKI

## Abstract

AXL receptor tyrosine kinase (RTK) inhibition presents a promising therapeutic strategy for aggressive tumor subtypes, as AXL signaling is upregulated in many cancers resistant to first‐line treatments. Furthermore, the AXL ligand growth arrest‐specific gene 6 (GAS6) has recently been linked to cancer drug resistance. Here, we established that challenging conditions, such as serum deprivation, divide AXL‐overexpressing tumor cell lines into non‐self‐sustaining and self‐sustaining subtypes in 3D spheroid culture. Self‐sustaining cells are characterized by excessive GAS6 secretion and TAM‐PDK‐RSK‐mTOR pathway activation. In 3D spheroid culture, the activation of the TAM‐PDK‐RSK‐mTOR pathway proves crucial following treatment with AXL/MET inhibitor BMS777607, when the self‐sustaining tumor cells react with TAM‐RSK hyperactivation and enhanced SRC‐AKT‐mTOR signaling. Thus, bidirectional activated mTOR leads to enhanced proliferation and counteracts the drug effect. mTOR activation is accompanied by an enhanced AXL expression and hyperphosphorylation following 24 h of treatment with BMS777607. Therefore, we elucidate a double role of AXL that can be assigned to RSK‐mTOR as well as SRC‐AKT‐mTOR pathway activation, specifically through AXL Y779 phosphorylation. This phosphosite fuels the resistance mechanism in 3D spheroids, alongside further SRC‐dependent EGFR Y1173 and/or MET Y1349 phosphorylation which is defined by the cell‐specific addiction. In conclusion, self‐sustenance in cancer cells is based on a signaling synergy, individually balanced between GAS6 TAM‐dependent PDK‐RSK‐mTOR survival pathway and the AXLY779/EGFR/MET‐driven SRC‐mTOR pathway.

**Abbreviations**
2D: two‐dimensional
3D: three‐dimensional
AKT: RAC‐α serine/threonine protein kinase
ATP: adenosine triphosphate
AXL: tyrosine protein kinase receptor UFO
DMSO: dimethyl sulfoxide
EGFR: epithelial growth factor receptor 1
eIF4B: eukaryotic translation initiation factor 4B
ELISA: enzyme‐linked immunosorbent assay
EMT: epithelial‐to‐mesenchymal transition
EOC: epithelial ovarian cancer
ERK5: mitogen‐activated protein kinase 7
ERK: extracellular signal‐regulated kinase
FBS: fetal bovine serum
GAB1: Grb‐associated binder 1
GAPDH: glyceraldehyde‐3‐phosphate dehydrogenase
GAS6: growth arrest‐specific gene 6
HGF: hepatocyte growth or scatter factor
HNSCC: head and neck sqamous cell carcinoma
HRP: horseradish peroxidase
IRS‐1: insulin receptor substrate 1
LC/MS: liquid chromatography–mass spectrometry
MERTK: proto‐oncogene tyrosine protein kinase MER
MET: MET proto‐oncogene or c‐MET
mTOR: mammalian target of rapamycin
nm: nanomolar
NMR: nuclear magnetic resonance
NSCLC: non‐small‐cell lung cancer
PBS: phosphate‐buffered saline
PCa: prostate cancer
PDK: phosphoinositide‐dependent kinase
PI3K: phosphatidylinositide 3‐kinase
PROS1: vitamin K‐dependent protein S
rpm: revolutions per minute
RSK: 90‐kDa ribosomal S6 kinase
RTK: receptor tyrosine kinase
SEM: standard error of mean
SHC: SHC‐transforming protein 1
siRNA: small interfering RNA
SRC: proto‐oncogene tyrosine protein kinase Src
TAM: TYRO3 and AXL and MERTK
TKI: tyrosine kinase inhibitor
TNBC: triple‐negative breast cancer
TYRO‐3: tyrosine protein kinase receptor 3
μm: micromolar

## 1 Introduction

Therapy resistance displays a major problem of targeted cancer therapies.

One promising therapeutic strategy to overcome this problem is AXL receptor tyrosine kinase (AXL) inhibition. AXL is upregulated in many cancers being resistant to first‐line treatments, and clinical trials with AXL inhibitors are ongoing in several cancers, including triple‐negative breast cancer (TNBC) and non‐small‐cell lung cancer (NSCLC) (Myers *et al*., 2016).

AXL is a member of the TYRO3, AXL, MERTK (TAM) family of receptor tyrosine kinases (RTKs). AXL activation occurs by binding of growth arrest‐specific gene 6 (GAS6), its only validated ligand (Stitt *et al*., 1995) (Varnum *et al*., 1995). AXL signaling stimulates phosphatidylinositide 3‐kinase/RAC‐α serine/threonine protein kinase (PI3K/AKT), extracellular signal‐regulated kinase (ERK) and p38 mitogen‐activated protein kinase cascades, the nuclear factor‐kappa B (NF‐κB) pathway, and signal transducer and activator of transcription signaling (Hafizi and Dahlback, 2006). Consequently, AXL signaling modulates biological processes, including invasion, angiogenesis, resistance to chemotherapeutics and targeted drugs, survival, and proliferation, which represent characteristics associated with malignancies (Linger *et al*., 2008).

Recently, publications point out that AXL‐activating ligand GAS6 is also linked to cancer drug resistance. This was shown by the application of high‐affinity AXL decoy receptor MYD1‐72, which improved the therapeutic index of doxorubicin in preclinical ovarian cancer models (Kariolis *et al*., 2017).

Further, epithelial–mesenchymal transition (EMT) is connected with GAS6 and the development of chemoresistance in epithelial ovarian cancer (EOC). RNA sequencing analysis revealed that TWIST1 expression resulted in upregulation of GAS6 and the activation of the AKT pathway. On the other side, knockdown studies demonstrated that the loss of GAS6 sensitized these cells to cisplatin treatment and suggest a potential mechanism of drug resistance in EOC (Roberts *et al*., 2016). Lee *et al*. could show that GAS6 regulates the cell cycle and apoptosis of prostate cancer (PCa) cells in response to docetaxel chemotherapy. GAS6 expression protected PCa cells from docetaxel‐induced apoptotic signaling, suggesting that GAS6 plays a significant role in the regulation of PCa cell survival during chemotherapy (Lee *et al*., 2016).

Independently of the AXL/GAS6 system, tumor cells develop a heterogeneous setup of resistance mechanisms to cancer drug treatments. For example, c‐Met (MET) and c‐Src (SRC) are alternative players in the puzzle of drug resistance mechanisms. MET contributes specifically to erlotinib resistance in head and neck squamous cell carcinoma (HNSCC) with activated SRC, where MET activation is dependent on SRC phosphorylation, providing an alternate survival pathway. The knockdown of endogenous SRC enhanced sensitivity to erlotinib, in contrast to treatment with HGF that induced MET phosphorylation and resulted in erlotinib resistance. Additionally, the level of endogenous phosphorylated SRC in HNSCC cell lines was also significantly correlated with erlotinib resistance (Stabile *et al*., 2013). Other research groups indicate that SRC‐mediated ERK reactivation may play a role in gefitinib resistance mechanism of EGFR‐mutant NSCLC cells, or that constitutively active SRC increased gemcitabine chemoresistance in contrast to dominant negative SRC that impaired gemcitabine chemoresistance of pancreatic adenocarcinoma cells (Ochi *et al*., 2014) (Duxbury *et al*., 2004).

The integrin β1/SRC/AKT signaling pathway was identified as a key mediator of acquired resistance to gefitinib and erlotinib treatment in NSCLC harboring EGFR‐activating mutations; in addition, SRC activity contributes to anoikis resistance of human osteosarcoma cells (SAOS). SRC was found to be upregulated in anoikis‐resistant SAOS cells, and pharmacological inhibition of its activity resulted in the restoration of anoikis sensitivity. Altogether, these studies indicated a SRC‐dependent activation of the PI3K/AKT pathway to be a critical component of survival pathways and drug resistances (Kanda *et al*., 2013) (Diaz‐Montero *et al*., 2006). Yori *et al*. could show that resistance to RTK blockade in breast cancer is often mediated by the activation of bypass pathways and that resistance to mTOR inhibitors results in phosphorylation and activation of AKT in SRC‐dependent manner. Dasatinib completely blocks this feedback activation, confirming convergence between SRC and the mTOR pathway. This suggests that combining mTOR and SRC inhibitors may circumvent resistance to targeted RTK therapies (Yori *et al*., 2014).

There is increasing evidence that drug‐resistant tumor cells significantly depend on 90‐kDa ribosomal S6 kinase (RSK) activation; thus, RSK emerges as another essential component in the resistance process. Therefore, a critical role is assigned to RSK downstream targets, including the oncogenic transcription factor YB‐1, which displays a significant feature of highly aggressive breast cancer as shown by Davies *et al*. (Davies *et al*., 2011). Other studies describe that in HER2‐amplified breast cancer, YB‐1 inhibition by targeting RSK repressed resistance to trastuzumab or that resistance to taxane in PCa therapy is linked to RSK and to its downstream target YB‐1 (Astanehe *et al*., 2012) (Shiota *et al*., 2014). Davies *et al*. and To *et al*. highlight the impact of RSK inhibition on the elimination of cancer stem cells and the inhibition of mammospheres in the context of drug resistance (Davies *et al*., 2015) (To *et al*., 2010).

Cancer stem cells have been defined as a small subset of cancer cells within a cancer that constitute a reservoir of self‐sustaining cells with the exclusive ability to self‐renew and to cause the heterogeneous lineages of cancer cells that comprise the tumor (Clarke *et al*., 2006). Recent studies have proposed that cancer‐initiating cells may be more resistant to irradiation than other cells in the population (Phillips *et al*., 2006). There are also reports that cancer‐initiating cells can be more resistant to chemotherapeutic drugs because of increased expression of antiapoptotic proteins or increased expression of the ATPase drug efflux pump ABCG2/5 (Frank *et al*., 2005).

This led us to the question whether self‐sustaining or cancer‐initiating cells could be identified by overcoming challenging conditions like serum deprivation and/or treatment with AXL/MET inhibitor BMS777607. Here, we present an easy feasible method to identify self‐sustaining cancer cells in 3D spheroid systems. Subsequently, we disclose the AXL/TAM RTKs in combination with their ligands GAS6/PROS1 as the decisive drivers of the PDK‐RSK‐mTOR survival pathway. The results lead to a model about the functionality of self‐sustaining cancer cells using PDK‐RSK survival pathway in combination with the SRC‐dependent proliferation pathway to finally benefit from the synergy effect of mTOR activation being responsible for drug resistance.

## 2 Materials and methods

### 2.1 Cell culture

Cells were cultured in a humidified incubator with 5% CO_2_ at 37° C. The human triple‐negative breast cancer (TNBC) cell line MDA‐MB231 was maintained in DMEM (No. 41965) supplemented with 1% sodium pyruvate and 10% FBS. The TNBC cell line Hs578T was cultured in RPMI 1640 medium (No. 31870) with 1% GlutaMAX and 10% FBS. The TNBC cell line MDA‐MB231‐D3H2LN (Caliper cell line) (Jenkins *et al*., 2005) was grown in MEM (No. 21090) supplemented with 1% sodium pyruvate, 1% GlutaMAX, 1% nonessential amino acids, and 10% FBS. All TNBC cell lines represent genetically the basal claudin‐low TNBC subtype. The human non‐small‐cell lung cancer (NSCLC) cell line NCI‐H292 (H292) was cultured in RPMI 1640 medium (No. 31870) with 1% GlutaMAX and 10% FBS and displays a mucoepidermoid pulmonary carcinoma. Cell culture media and supplements were purchased from Gibco (Thermo Fisher Scientific, Darmstadt, Germany).

### 2.2 Three‐dimensional (3D) spheroid culture

Matrigel basement membrane matrix (BD Biosciences—Corning, No. 354234; Kaiserslautern, Germany) was diluted at a concentration of 3 mg·mL^−1^ in cell line‐corresponding serum‐free medium. Precooled Matrigel was pipetted into the wells of 96‐well plates (80 μL) or 6‐well plates (2.4 mL) for cell viability assays and 3D cell lysate preparations, respectively. The Matrigel polymerized at least 16 h at 37° C. Cells in serum‐free medium were seeded in concentrations of 7000 per 120 μL (96‐well plates) or 2.1 × 10^5^ per 3.6 mL (6‐well plates) on top of the solid Matrigel. Specific inhibition of GAS6 and PROS1 was performed by the addition of αGAS6 (AB885) and αPROS1 (AF4036) at the time of cell seeding. Both antibodies were purchased from R&D Systems (Abingdon, UK).

### 2.3 Tyrosine kinase inhibitors and cell treatment

BMS777607 was purchased from ShangHai Biochempartner Co., Limited, Wuhan, China (Cas No.:1196681‐44‐3) with a purity > 98%, which passed an independent quality control check by LC/MS and NMR analysis at the Lead Discovery Center GmbH, Dortmund, Germany. MPCD84111, sunitinib, dasatinib, and imatinib were obtained from Vichem Chemie Research Ltd. (Budapest, Hungary). MPCD84111 is patented under application example 12 from WO2011045084. All inhibitors were dissolved in DMSO (Sigma Aldrich) and stored at room temperature in 10 mm or 5 mm stock solutions.

Cells were cultivated in petri dishes (2D) or 6‐well plates (3D) and treated with TKIs previously prediluted at micromolarity with serum‐free medium. For the treatment of 3D spheroids in 96‐well plates, 1 : 2 dilution rows of the compounds were prepared. For homogenous distribution of TKIs in 6‐well MG assays, 1 mL cell supernatant was removed to polypropylene tube. TKI was added to the supernatant. After mixing, the supernatant was retransferred immediately to the cells.

### 2.4 Cell viability assay

Viability of cells was determined by ATP quantitation using CellTiter‐Glo Luminescent Cell Viability Assay (Promega, Mannheim, Germany).

Cells grown as 3D spheroids on Matrigel in 96‐well plates were mixed as a whole with 100 μL CellTiter‐Glo reagent per well. The luminescence signal was measured after 20 min using a TECAN Ultra Evolution multimode microplate reader (Tecan Group Ltd., Männedorf, Switzerland).

### 2.5 Cell extracts of 3D spheroids

Supernatant of 3D cultured cells was quantitatively removed. Six‐well plates were placed on ice before adding 1 mL of ice‐cold PBS per well. Cells and Matrigel were transferred to precooled Eppendorf cups. Wells were washed twice with 1 mL ice‐cold PBS for the complete transfer of cells. Cells were centrifuged for 2 min at 10 000 rpm (8000 ***g***) in Eppendorf centrifuge (Eppendorf AG, Hamburg, Germany). Supernatants containing resolved Matrigel were removed and cell pellets were suspended in ice‐cold PBS. After centrifugation for 1 min at 10 000 rpm, supernatants with residual Matrigel were quantitatively removed. Pelleted cells were lysed in lysis buffer for 15 min.

### 2.6 Western blot analysis

Cell lysis and western blot analysis were performed as previously described (Penzes *et al*., 2014; Torka *et al*., 2014). Primary antibodies pAKT S473 (No. 4060), p‐eIF4B S422 (No. 3591), pEGFR Y1173 (No. 4407), pERK 1/2 T202/Y204 (No. 4370), pERK5 T218/Y220 (No. 3371), pGAB1 Y627 (No. 3233), pIRS‐1 S612 (No. 3203), pIRS‐1 Y895 (No. 3070), pMET Y1234 (No. 3077), pMET Y1349 (No. 3133), pPDK S241 (No. 3438), pRSK S380 (No. 11989), pSHC Y240 (No. 2434), pSRC Y416 (No. 6943), p‐mTOR S2448 (No. 2971), AXL (No. 8661), MER (No. 4319), TYRO3 (No. 5585), ACTIN‐HRP (No. 12620), and GAPDH (No. 8884) were purchased from Cell Signaling (New England Biolabs GmbH, Frankfurt am Main, Germany). Phospho‐AXL Y779 (AF2228), anti‐GAS6 (AB885), and anti‐PROS1 (AF4036) antibodies were purchased from R&D (R&D Systems). Phospho‐p70S6K T229 (No. 12021) was purchased from SAB Biotech (Antibodies‐Online GmbH, Aachen, Germany).

### 2.7 Immunoprecipitation

Immunoprecipitation was performed as previously described (Torka, Penzes *et al*., 2014). The home‐made clone 4G10 was used as antibody for phospho‐tyrosine precipitation in the concentration of 1** **μg per 250 μg cell extract. Subsequently, the AXL antibody (No.8661, Cell Signaling, New England Biolabs GmbH) was used for western blot analysis.

### 2.8 Enzyme‐linked immunosorbent assay (ELISA)

Supernatants were centrifuged for 5 min at 2000 rpm (700 ***g***). GAS6 detection was performed with 10 μL and PROS1 detection with 100 μL supernatant.

The GAS6‐specific ELISA kit (R&D Systems, No. DY885) was used for the quantification of GAS6 according to the manufacturer's protocol with the following modifications: Monoclonal capture antibody of the kit was replaced by the monospecific αGAS6 antibody AB885 (R&D Systems). After binding of the biotinylated GAS6‐specific detection antibody, we used an alkaline phosphatase‐conjugated streptavidin SA110 (Millipore, Darmstadt, Germany) (1 : 4000) with fluorometric detection of the alkaline phosphatase by AttoPhos substrate set (Roche Diagnostics GmbH, Mannheim, Germany) (100 μL per well). PROS1 detection was performed in analogy to the GAS6 detection with αPROS1 antibody AF4036 (R&D Systems) and biotinylated αPROS1 antibody LS‐C298521 (LSBio, Eching, Germany). The fluorometric signal was quantified after 30 min (GAS6) or 2 h (PROS1) at a wavelength of 430/560 nm using a TECAN Ultra Evolution multimode microplate reader (Tecan Group Ltd).

### 2.9 Microscopy

Phase‐contrast images were captured on an Axiovert 300 microscope (Carl Zeiss AG, Oberkochem, Germany) using MetaMorph (Molecular Devices, Sunnyvale, CA, USA).

### 2.10 Statistical data analysis

All assays were performed at least in triplicate. Mean values and SEM are shown.

The statistical analysis was performed by the application of an unpaired, two‐tailed *t*‐test or a one‐way ANOVA in combination with Bonferroni multiple comparison post‐test using graphpad prism 5 (GraphPad Software, Inc, La Jolla, CA, USA). Differences with *P < 0.05 were considered as statistically significant.

## 3 Results

### 3.1 Autocrine GAS6 triggers RSK phosphorylation in starvation conditions

**Figure 1:**
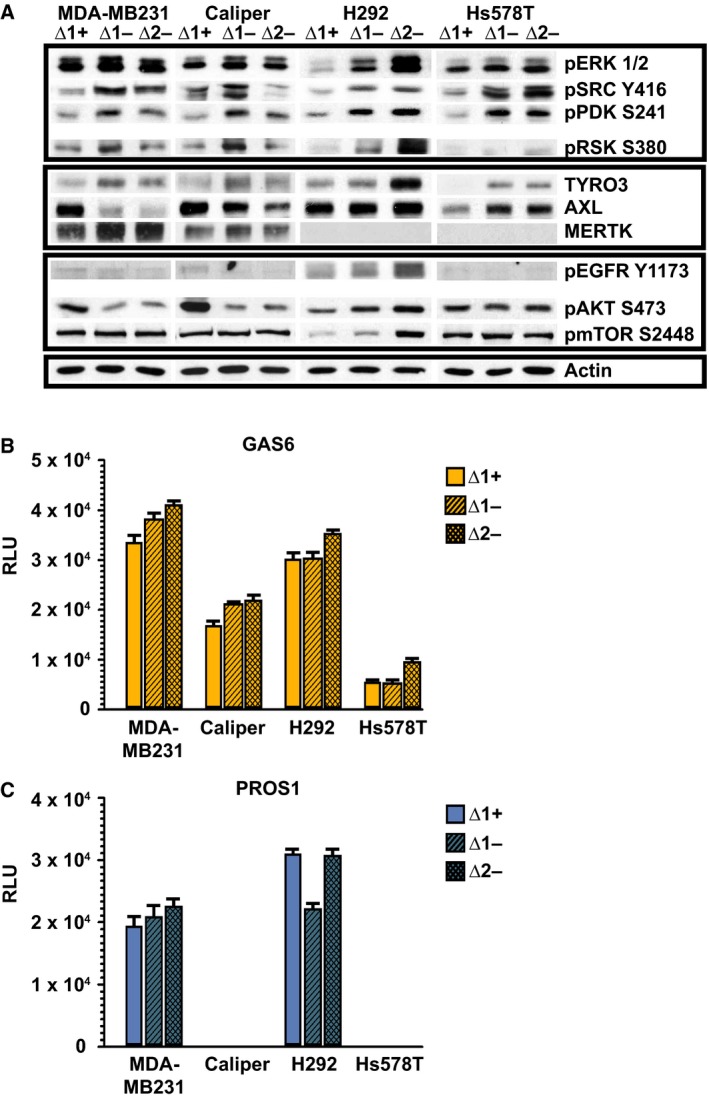
Serum deprivation induces RSK‐driven mTOR activation and correlates with autocrine GAS6 level. (A) Western blot analysis of 2D cultivated MDA‐MB231, Caliper, H292, and Hs578T cells that are shown being either cultivated for 24 h with FBS (Δ1+) or without FBS for 24 (Δ1−) and 48 h (Δ2−). The lack of growth factors activates ERK T202/Y204 and SRC Y416 and specifically in H292 EGFR Y1173 and AKT S473 phosphorylation. TAM receptor analyses were performed to substantiate a direct link to the PDK‐RSK‐dependent mTOR activation. ACTIN served as loading control for all western blots. A representative sample of multiple biological replicates is displayed in the bottom line. (B and C) High autocrine GAS6, and not PROS1, matches with pRSK S380 activation in tumor cells. Supernatants of 2D cultivated tumor cells were analyzed by GAS6 ELISA (B) and PROS1 ELISA (C). The results represent the average of fourfold determination.

We aimed to compare the impact of challenging conditions on major intracellular signaling cascades of AXL RTK‐overexpressing triple‐negative breast cancer (TNBC) and non‐small‐cell lung cancer (NSCLS) cell lines, namely MDA‐MB231, Caliper, Hs578T, and H292. Therefore, we used serum deprivation conditions and subsequently examined the phosphorylation status of pERK T202/Y204, pSRC Y416, and pPDK S241 after 24 and 48 h of starvation (Fig. 1A and Fig. S2). Serum deprivation enhanced the phosphorylation of ERK, SRC, and PDK consistently in all cell lines. We additionally determined a significant impact on pRSK S380 phosphorylation in MDA‐MB231, Caliper, and H292 in contrast to hardly detectable RSK levels in Hs578T cells. RSK is a known upstream kinase and activator of the mTOR complex and thus responsible for the stabilization of mTOR S2448 phosphorylation. We observed AKT‐independent mTOR stabilization in MDA‐MB231 and Caliper cells as indicated by decreased pAKT S473 levels. On the other hand, H292 cells displayed an increasing AKT phosphorylation accompanied by enhanced pEGFR Y1173 phosphorylation, indicating that serum starvation activates their EGFR‐AKT pathway. Subsequently, we focused on the upstream RTKs being responsible for pRSK S380 activation, and thus, we analyzed the TAM RTK family based on the fact that the selected cell lines commonly express high levels of autocrine GAS6 (Fig. 1B). MDA‐MB231 and H292 cells secrete autocrine PROS1 likewise as quantified by PROS1 ELISA (Fig. 1C). The expression analysis of TAM RTKs revealed that AXL and TYRO3 are expressed in all four cell lines, in contrast to MERTK that is restricted to MDA‐MB231 and Caliper cells. In analogy of activated RSK, we observed an increased expression of TYRO3 and MERTK, while AXL expression surprisingly decreased in FBS‐starved MDA‐MB231 and Caliper cells. In H292 cells, we determined an enhanced TYRO3 and AXL expression in analogy of pRSK S380 activation.

These results indicate that challenging conditions induced by serum deprivation lead to RSK phosphorylation based on high autocrine GAS6 and cell‐specific TAM RTK expression in the respective cell lines.

### 3.2 The PDK‐RSK‐mTOR pathway is enhanced by activation of the TAM–GAS6 system after MET Y1234 inhibition in MDA‐MB231 and Caliper cells

**Figure 2:**
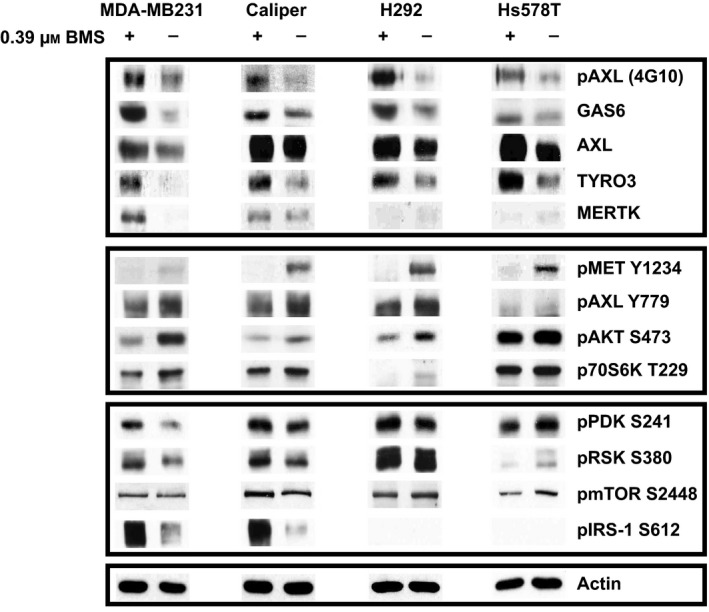
TAM‐GAS6 and PDK‐RSK‐mTOR activation after MET Y1234 inhibition in MDA‐MB231 and Caliper cells. 2D cultivated MDA‐MB231, Caliper, H292, and Hs578T cells were treated for 24 h with 0.39 μm BMS777607 without FBS. Cell lysates of treated and control cells were analyzed by western blot. A significant pathway switch is shown in Caliper and MDA‐MB231 where low‐dose BMS777607 treatment reduces the AKT‐driven pathway (middle section) but induces the PDK‐RSK‐mTOR‐driven pathway (lower section) by the activation of AXL, the TAM family and its ligands (upper section). The pathway switch is connected to an increase in pIRS‐1 S612. An activated TAM‐PDK‐RSK pathway is also determined in H292, whereas Hs578T TAM receptor activation is not connected to the PDK‐RSK‐driven mTOR pathway. ACTIN served as loading control for all western blots. A representative sample of multiple biological replicates is displayed in the bottom line.

We examined the effects of the AXL/MET inhibitor BMS777607 on the intrinsic potential of TAM family members to counteract the effects of AXL/MET inhibition after exposure to 0.39 μm low‐dose BMS777607 for 24 h. Figures 2, S3 and S4 depict the effect of pMET Y1234 inhibition, which subsequently decreases AKT S473 and p70S6K T229 phosphorylation. Interestingly, we observed a strong induction of AXL phosphorylation by immunoprecipitation when using tyrosine antibody (4G10), and increased expression of autocrine GAS6, AXL, and TYRO3 after BMS777607 treatment in all cell lines. In analogy to the challenging conditions displayed in Fig. 1A, we could proof an activated PDK‐RSK‐mTOR pathway in MDA‐MB231 and Caliper cells with a corresponding induction of MERTK, additionally (Fig. 2 and Fig. S3). Furthermore, an enhanced IRS‐1 S612 phosphorylation was observed in both cell lines. The IRS‐1 S612 phosphorylation site is considered to be a negative regulator of the PI3K pathway by prohibiting RTK binding to PI3K, as previously described (Andreozzi *et al*., 2004). Noteworthy is that AXL Y779 phosphorylation does not correlate with overall AXL phosphorylation detected after tyrosine immunoprecipitation. Therefore, we infer that pAXL Y779 is not involved in the PDK‐RSK activation process.

In summary, we conclude that GAS6‐TAM ligand binding results in PDK‐RSK‐mTOR pathway activation in MDA‐MB231 and Caliper. This implicates the relationship of TAM receptors and RSK activation. The induction of AXL, TYRO3 as well as GAS6 expression in H292 cells leads to pRSK S380 and p‐mTOR S2448 stabilization, despite AKT S473 inhibition. This is in contrast to the situation in Hs578T cells where the RSK‐mTOR pathway is not responsive.

### 3.3 GAS6‐dependent RSK activity corresponds with 3D survival

**Figure 3:**
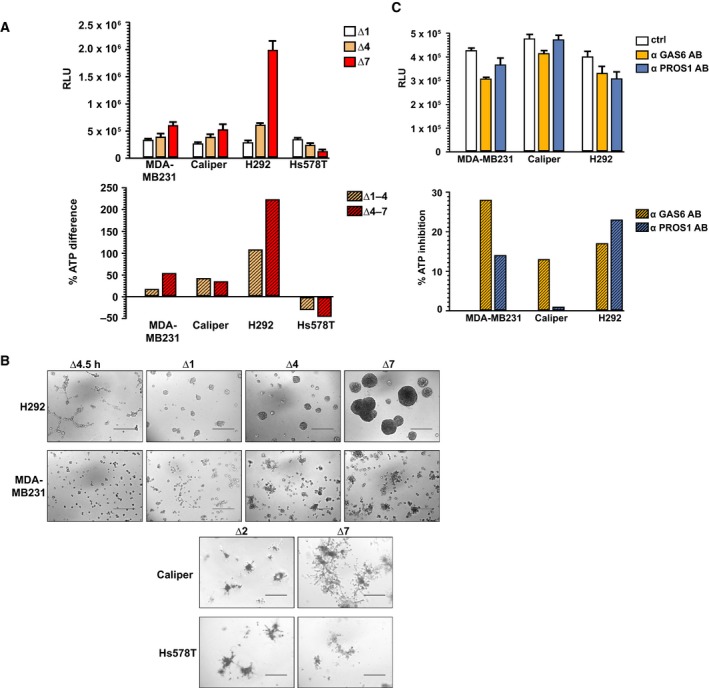
GAS6‐dependent RSK activity corresponds with 3D survival. (A) MDA‐MB231, Caliper, H292, and Hs578T cells were cultivated in 3D Matrigel assays without FBS. ATP measurements were taken after one (Δ1), four (Δ4), and 7 days (Δ7) of cultivation. The percentage increase in ATP within each 3‐day period (Δ1‐4 and Δ4‐7) was calculated for MDA‐MB231, Caliper, and H292 cells, in contrast to Hs578T cells exhibiting a decrease in ATP content. (B) Images of cultivated cells demonstrate the extraordinary high proliferation and phenotypic behavior of H292, visible proliferation of MDA‐MB231 and Caliper cells, and the lack of proliferation of Hs578T cells in 3D. Calibration bars correspond to 200 μm. (C) MDA‐MB231, Caliper, and H292 cells were cultivated in 3D Matrigel assay for 1 day in the presence of αGAS6 (10 μg·mL ^−1^) and αPROS1 (5 μg·mL ^−1^) antibody. ATP measurements were taken to quantify the inhibitory effect.

In order to grasp the benefit of TAM and RSK activation, we performed time course experiments with the indicated cell lines in 3D Matrigel assays in starving conditions. Cell vitality measurement revealed that ATP content gradually increased in MDA‐MB231, Caliper, and H292 in contrast to Hs578T cells (Fig. 3A). H292 cells displayed an extraordinary increase in ATP content, which is reflected by the phenotype of big and compact spheroids after 7 days (Fig. 3B). The capability of proliferation in 3D is in accordance with the capability of RSK activation. Therefore, we postulate that RSK activation is a prerequisite of the tumor cells for survival and proliferation in starving conditions. Moreover, the extremely high proliferation rate of H292 in 3D may be attributable to an autocrinely activated EGFR as ascertained in 2D cultivated cells (Fig. 1A). This is in sharp contrast to Hs578T cells lacking RSK activation and survival in 3D culture, accordingly. Subsequently, we analyzed the influence of autocrine GAS6 and PROS1 on proliferation. Therefore, we cultivated the tumor cells as 3D Matrigel spheroids in the presence of αGAS6 or αPROS1 antibody (Fig. 3C). We observed that the analyzed cell lines react to GAS6 inhibition with a reduction of ATP content of 14 to 28%. Blocking of PROS1 by antibody reduced ATP in MDA‐MB231 and H292 cells likewise. The absence of PROS1 ligand in Caliper cells (Fig. 1C) is accompanied by the lack of efficiency of αPROS1 antibody to inhibit the proliferation of 3D spheroids.

In summary, the ATP inhibition by αGAS6 and αPROS1 antibodies reveals the ligand‐driven survival and proliferation of tumor cells in 3D. Moreover, it underlines the impact of TAM RTKs on proliferation in 3D.

### 3.4 Identification of SRC‐dependent RTK phosphorylation sites by dasatinib treatment

**Figure 4:**
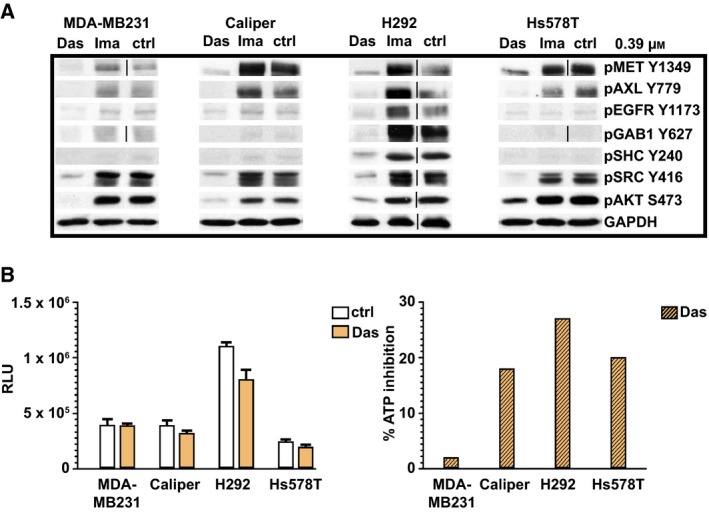
Impact of SRC‐dependent RTKs on proliferation. (A) Influence of SRC‐dependent RTK phosphorylation sites was identified by dasatinib treatment. 2D cultivated cells were treated with 0.39 μm dasatinib for 1 day. Western blot analyses revealed reduced phosphorylation of pMET Y1349, pAXL Y779, and pEGFR Y1173 in consequence of SRC inhibition. Decreased RTK phosphorylation results in reduced AKT S473 phosphorylation. Inhibition of pEGFR Y1173 in H292 is accompanied by pGAB1 Y627 and pSHC Y239/240 inhibition. Imatinib has no impact on pAKT S473. This is in analogy to the stability of RTK phosphorylation. (B) ATP measurement of 3D cultivated cells after 2 nm dasatinib treatment. Dasatinib decreased the ATP content of Caliper, H292 as well as Hs578T in contrast to MDA‐MB231 cells. Composed western blots are indicated by black vertical lines.

We were able to detect enhanced pSRC Y416 phosphorylation in all cell lines after 24 h of FBS deprivation in 2D culture conditions (Fig. 1A). The following experiments were performed to identify the influence of SRC on RTK phosphorylation. The inhibition of SRC by dasatinib figured out that pAXL Y779, pMET Y1349, and pEGFR Y1173 phosphorylations depend on SRC activity. Moreover, the dominant activation of pGAB1 Y627 and pSHC Y239/240 implies that in H292 cells, a characteristic mutual upregulation between pSRC Y416 and pEGFR Y1173 phosphorylation exists (Sato *et al*., 2000). Additionally, we proved a reduced pAKT S473 phosphorylation by inhibition of upstream RTKs in a SRC‐dependent manner. This is in contrast to the imatinib treatment which is a non‐SRC‐specific compound being used as a control inhibitor (Fig. 4A and S5). We treated cells with 2 nm dasatinib in 3D cultures and determined the cell viability by ATP measurement. As shown in Fig. 4B, ATP content decreases by 18 to 28%, with the exception of MDA‐MB231 cells, when SRC phosphorylation is inhibited. In consequence, Caliper and H292 cells slow down their proliferation rate, whereas Hs578T exhibits an accelerated loss of cell viability with regard to their proliferation profile. The lower SRC dependency of MDA‐MB231 in 3D is reflected by the lower impact of dasatinib on cell viability (Fig. 4B).

### 3.5 Pathway synergy of RSK and SRC is responsible for drug resistance

**Figure 5:**
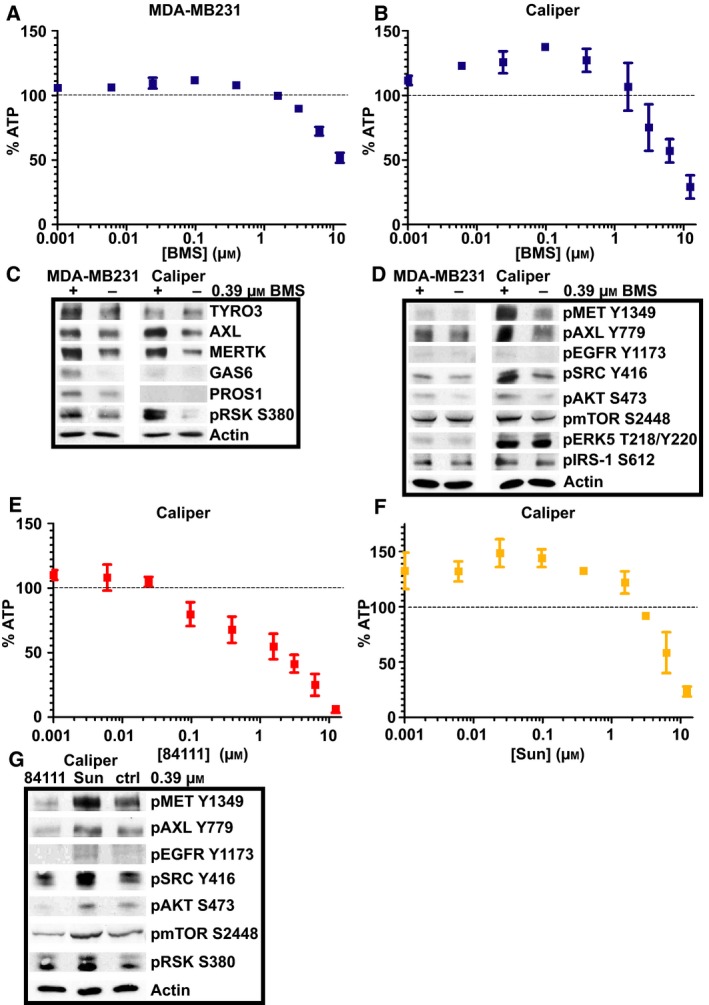
Synergy of RSK and SRC pathway activating RTKs induces ATP in consequence of low‐dose BMS777607 treatment in Caliper and MDA‐MB231. (A  +  B) Cells were cultivated in 3D in the presence of increasing BMS777607 concentrations in a range between 0.001 and 12.5 μm. ATP measurements were taken after 3 days. DMSO control values were set to 100% and indicated by a doted horizontal line. Nanomolar concentrations of BMS777607 increase significantly the ATP content of Caliper cells and to a lower extent of MDA‐MB231 cells, in contrast to micromolar concentrations. Percentages of ATP inhibition were calculated from at least three independent experiments. (C) MDA‐MB231 and Caliper cells were treated with 0.39 μm BMS777607 for 3 days. The western blot analyses display the induction of RSK S380 phosphorylation in both cell lines compared to untreated cells. MDA‐MB231 cells activated all three TAM members, autocrine GAS6 and PROS1, whereas Caliper recruited AXL, MERTK, and GASA6 in consequence of BMS777607 treatment. (D) Further western blot analyses show an enhancement of SRC and SRC‐dependent MET Y1349 and AXL Y779 phosphorylation with a slightly enhanced AKT S473 signal in both cell lines. A significant ERK5 phosphorylation is only detectable in Caliper cells in accordance with a stronger pMET Y1349 and pSRC Y416 activation compared to MDA‐MB231 cells. (E and F) Caliper cells were cultivated in 3D in the presence of 0.001–12.5 μm of 84 111 and sunitinib. ATP measurements were taken after 3 days. DMSO control values were set to 100% and indicated by a doted horizontal line. A significant ATP increase was induced by nm concentrations of sunitinib in contrast to nm concentrations of 84 111. An inhibition of ATP by sunitinib was detectable at concentrations ≥ 1.56 μm and at concentrations ≥ 0.097 μm by 84 111. (G) Caliper cells were treated for 3 days either with 0.39 μm 84 111 or with sunitinib. Western blot analyses show the RSK and SRC‐dependent pathway activation by sunitinib compared to untreated cells. 84 111 reduced AKT‐mTOR phosphorylation due to inhibition of SRC as well as MET Y1349 and AXL Y779 phosphorylation, but had no effect on RSK phosphorylation.

BMS777607 treatment (0.39 μm) activated the PDK‐RSK‐mTOR pathway of MDA‐MB231 and Caliper cells in 2D culture conditions. BMS777607 treatment triggered the expression of the TAM family members and autocrine GAS6, while AKT S473 phosphorylation decreased (Fig. 2A). We determined the impact of BMS777607 treatment in 3D culture conditions, accordingly. Therefore, we performed ATP assays of cells treated for 3 days with increasing concentrations of BMS777607. High BMS777607 concentrations (μm) reduced ATP content in MDA‐MB231 and Caliper cells, but lower concentrations (nm) increased the ATP content when compared to DMSO‐treated control, as shown in Fig. 5A,B. Therefore, it became evident that Caliper cells display a more pronounced ATP induction in nm range than MDA‐MB231 cells. Subsequently, we analyzed the protein expression and phosphorylation status of 3D cultivated cells after treatment with 0.39 μm BMS777607 (Fig. 5C,D and S6). Firstly, treatment with BMS777607 resulted in pRSK S380 stimulation, which was in analogy to 2D results. Secondly, MDA‐MB231 cells are capable of elevating protein levels of TYRO3, AXL, and MERTK accompanied by the expression of the corresponding ligands, namely GAS6 and PROS1. Caliper cells increased the expression of AXL, MERTK, and GAS6, but not of TYRO3 and PROS1. Thirdly, we determined a BMS777607‐induced activation of pSRC Y416, pMET Y1349, and pAXL Y779 in both 3D‐treated cell lines. Therefore, Caliper cells displayed a stronger induction of SRC‐dependent receptor phosphorylation combined with pERK5 than MDA‐MB231 cells (Fig. 5D). A more detailed analysis showed that pAKT S473 became activated by 0.39 μm BMS777607, which was accompanied by pIRS‐1 S612 stability in both 3D lysates. This implies that the PI3K pathway is not inhibited in 3D in contrast to 2D cultured cells treated with BMS777607. In summary, the autocrine activation of the SRC‐AKT‐dependent RTK phosphorylation in parallel with TAM‐RSK activation induces an effective resistance mechanism to low‐dose TKI treatment in 3D culturing conditions. The BMS777607 treatment of MDA‐MB231 and Caliper reveals that a bidirectionally activated mTOR is crucial to boost ATP content. Therefore, Caliper cells have the capacity to activate mTOR in TYRO3‐ and PROS1‐independent fashion.

In the next step, we counterchecked the impact of SRC inhibition. Therefore, we treated Caliper cells with MPCD84111, which was characterized as an efficient inhibitor for AXL‐MET‐SRC by Penzes *et al*. (2014), and sunitinib, which was characterized as a multitargeted TKI, but without the impact on MET or SRC inhibition (Penzes *et al*., 2014). We performed ATP assays 3 days after treatment of cells with increasing concentrations of MPCD84111 or sunitinib (Fig. 5E,F). High MPCD84111 and sunitinib concentrations (μm) reduced ATP content in Caliper cells, but low sunitinib concentrations (nm) increased the ATP content in contrast to MPCD84111. We speculate that the capacity of MPCD84111 to block MET and SRC phosphorylation prohibits the resistance reaction to AXL inhibitors. This is in clear contrast to the reaction of Caliper cells to sunitinib treatment, leading to an increase in ATP content of 40–50% when exposed to 24  to 390 nm concentrations. Further, analysis of signaling pathways revealed that pAXL Y779, pMET Y1349, and pEGFR Y1173 were inhibited by the SRC targeting compound MPCD84111 (Fig. 5G and S7). Consequently, pAKT S473 was inhibited as well. Conversely, treatment of Caliper cells with 0.39 μm sunitinib promoted phosphorylation of AKT S473 based on the increase in SRC‐dependent RTK phosphorylation. Moreover, treatment with 0.39 μm sunitinib led to the induction of pRSK S380.

Hence, p‐mTOR S2448 becomes bidirectionally activated after sunitinib treatment and subsequently ATP is triggered in analogy to BMS777607. This is in sharp contrast to the treatment with compound MPCD84111, which prevents the SRC‐dependent resistance mechanism as well as the activation of RSK.

### 3.6 SRC activity is fueled by HGF/MET in Caliper cells

**Figure 6:**
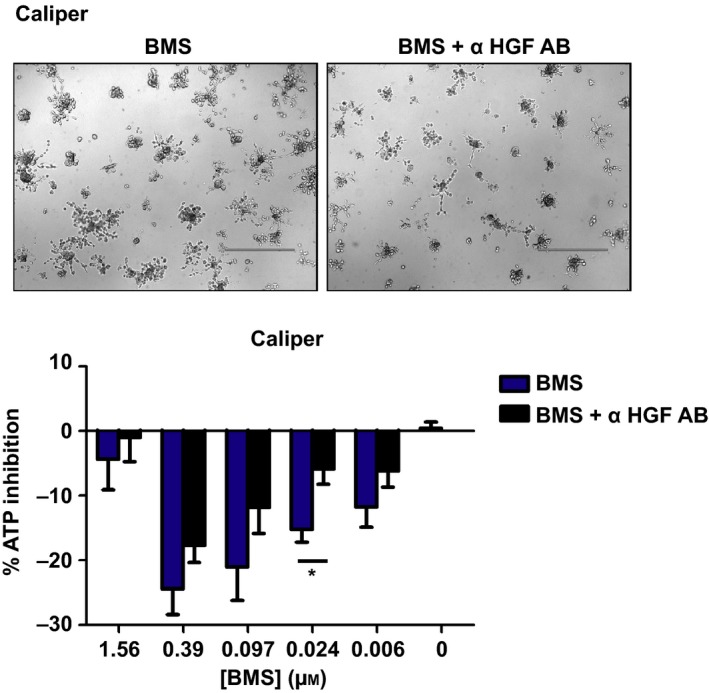
SRC activity in Caliper is fueled by HGF/MET. Caliper cells were cultivated in 3D Matrigel assay for 3 days in the presence of BMS777607 and αHGF antibody. The potent ATP induction obtained by BMS777607 treatment was reduced when HGF‐specific antibody was added in concentrations of 0.4 μg·mL ^−1^. Statistical significance was achieved using unpaired, two‐tailed *t*‐test (*P < 0.05). Images show the phenotypic impact of a prohibited resistance mechanism due to combinatorial treatment with 0.39 μm BMS777607 and αHGF antibody.

Finally, we examined whether the significant ATP increase can be ascribed to an autocrine loop of HGF in Caliper cells when being treated with nm concentrations of BMS777607. Based on this theory, a significant decrease in cell viability should be achieved by blocking HGF with antagonistic antibodies. We proved this assumption by simultaneous application of nm BMS777607 concentrations and αHGF‐specific antibodies on Caliper cells. The ATP quantification shown in Fig. 6 proves an attenuated resistance reaction being achieved by αHGF‐specific antibody. Additionally, we could show that HGF blocking is accompanied by a decreased cell invasion efficacy of Caliper 3D spheroids.

### 3.7 mTOR is primarily triggered by SRC‐dependent pathway in H292 or by GAS6‐RSK‐dependent pathway in MDA‐MB231 cells

**Figure 7:**
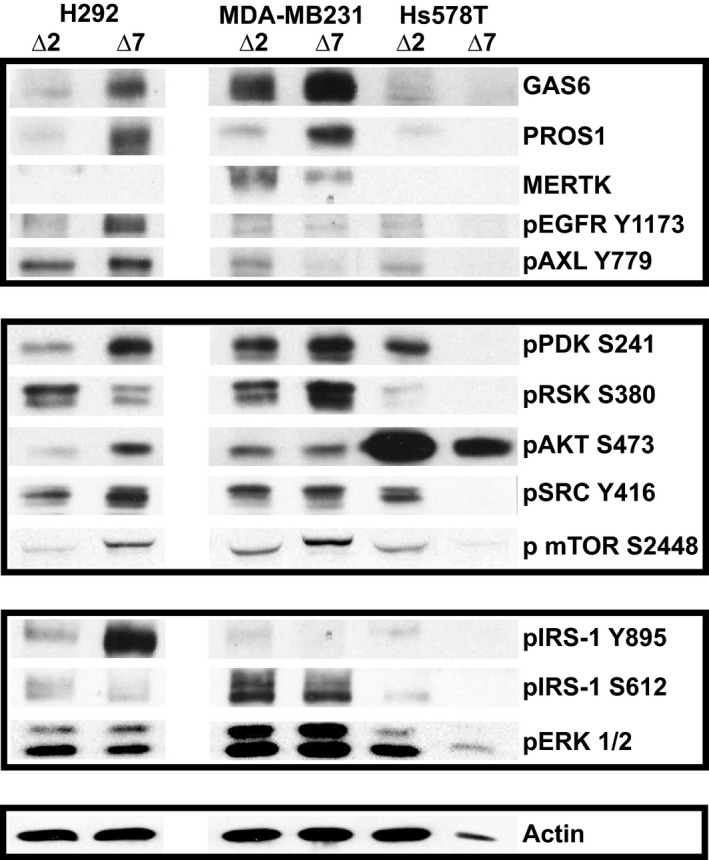
mTOR is triggered by SRC‐dependent pathway in H292 or by GAS6‐dependent RSK pathway in MDA‐MB231 cells. H292, MDA‐MB231, and Hs578T cells were cultivated in 3D without FBS. Cell lysates were prepared at day 2 as well as day 7 and subsequently analyzed by western blot. The representative western blot displays identical exposure times for each cell line. Prolonged cultivation of H292 and MDA‐MB231 cells shows activated mTOR which results from different activated pathways. Hs578T cells at day 7 display reduced ACTIN and mTOR but still elevated AKT levels compared to H292 and MDA‐MB231 cells. ACTIN served as loading control for all western blots. A representative sample of multiple biological replicates is displayed in the bottom line.

MDA‐MB231 and H292 cells express autocrine GAS6 as well as PROS1 and are sensitive to PROS1 and GAS6 inhibition by antagonistic antibodies in 3D (Figs 1B and 3C). However, the cell lines differ in the activation of pEGFR Y1173, which is highly phosphorylated in H292, whereas elevated expression of MERTK displays a characteristic feature of MDA‐MB231 cells (Figs 1A and 7). The analysis of 3D cell lysates prepared at day 2 and day 7 revealed that the strong proliferation rate of H292 can be ascribed to an autocrine activated pEGFR Y1173 and pAXL Y779. In consequence, activation of the PDK‐SRC‐AKT‐dependent pathway appears mainly responsible for the increase in p‐mTOR S2448 in H292 cells. Conversely, the increase in mTOR in MDA‐MB231 is triggered by GAS6‐RSK activation (Fig. 7 and S8). H292 cells display a higher SRC dependency compared to MDA‐MB231 cells which could be highlighted by the influence of SRC inhibitor dasatinib. As shown in Fig. 4B, dasatinib has a more pronounced effect on cell viability inhibition of H292 cells (28%) compared to MDA‐MB231 cells (3%). Consequently, the activation of p‐mTOR S2448 is attainable in two different ways dependent on the individual outfit and capacities of the tumor cells to activate these pathways by upstream kinases. This is in sharp contrast to Hs578T cells where a distinct AKT signal fails to activate p‐mTOR S2448 and an activation of pRSK S380 is not promoted in this cell line (Fig. 7 and S9). In summary, H292 and MDA‐MB231 possess the capability to activate p‐mTOR S2448 during prolonged stress condition. This is the decisive advantage of these cell lines for survival and proliferation. Therefore, long‐time p‐mTOR activation is individually attained either by a dominant SRC‐AKT pathway like in H292 or by a dominant GAS6‐RSK activation like in MDA‐MB231 cells. Moreover with the involvement of pIRS‐1 Y895 in H292 and pIRS‐1 S612 in MDA‐MB231, a switch point may exist to regulate cells individually in the direction of AKT or RSK dependent on the availability of pEGFR Y1173 like in H292 or an extraordinarily high GAS6 expression in the presence of MERTK like in MDA‐MB231.

### 3.8 SRC‐mTOR activation without RSK increase is not sufficient to boost ATP in consequence of low‐dose BMS777607 treatment

**Figure 8:**
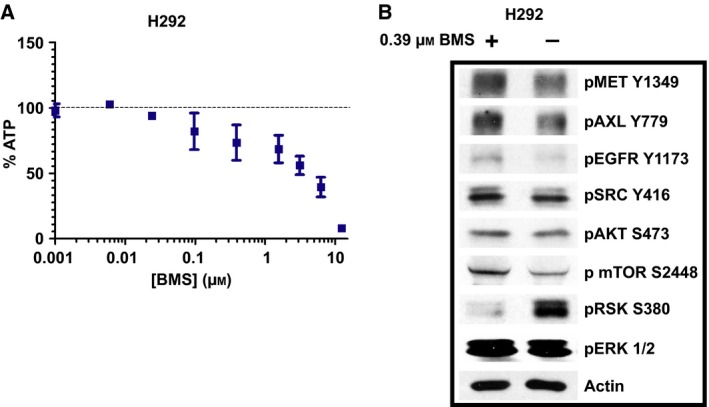
SRC‐mTOR activation without RSK increase is not sufficient to boost ATP in consequence of low‐dose BMS777607 treatment. (A) H292 cells were cultivated in 3D in the presence of 0.001 – 12.5 μm concentrations of BMS777607. ATP measurements were taken after 3 days. ATP was clearly decreased by concentrations ≥ 0.24 μm. Percentages of ATP inhibition were calculated from at least three independent experiments. (B) H292 cells were treated for 3 days with 0.39 μm BMS777607. The western blot analysis displays an enhancement of pSRC Y416 and SRC‐dependent pEGFR Y1173, pMET Y1349, and pAXL Y779 phosphorylation. H292 exhibits activated mTOR S2448 phosphorylation, while RSK S380 phosphorylation decreased.

The increased ATP content of Caliper cells being exposed to low dose of BMS777607 is a consequence of the dual activation of RSK and AKT (Fig. 5C,D). Unexpectedly, treatment of H292 cells with 0.39 μm BMS777607 led to decreased ATP amounts (Fig. 8A). In order to figure out the reason for the difference between Caliper and H292 cells, we further analyzed signaling cascades in more detail by western blot. We observed that BMS777607 treatment induced p‐mTOR S2448 phosphorylation by the activation of pAXL Y779, pMET Y1349, and pEGFR Y1173 but not by an increase in RSK S380 phosphorylation. This indicated a clear difference between Caliper and H292 cells. This result confirmed the finding of an increased SRC‐mTOR dependency of H292 in 3D (Fig. 8B and S10). Additionally, it displayed that the potential of an enhanced mTOR is not sufficient to compensate an ATP decrease as a consequence of AXL inhibitor treatment. Consequently, RSK activation seems to be crucial for compensation or feedback mechanism toward AXL TKI treatment. In summary, 3D cultured H292 strongly benefits from SRC‐dependent AXLY779/MET/EGFR RTKs activation after nm AXL TKI treatment. However, this is accompanied by a decreased ability to activate RSK.

**Figure 9:**
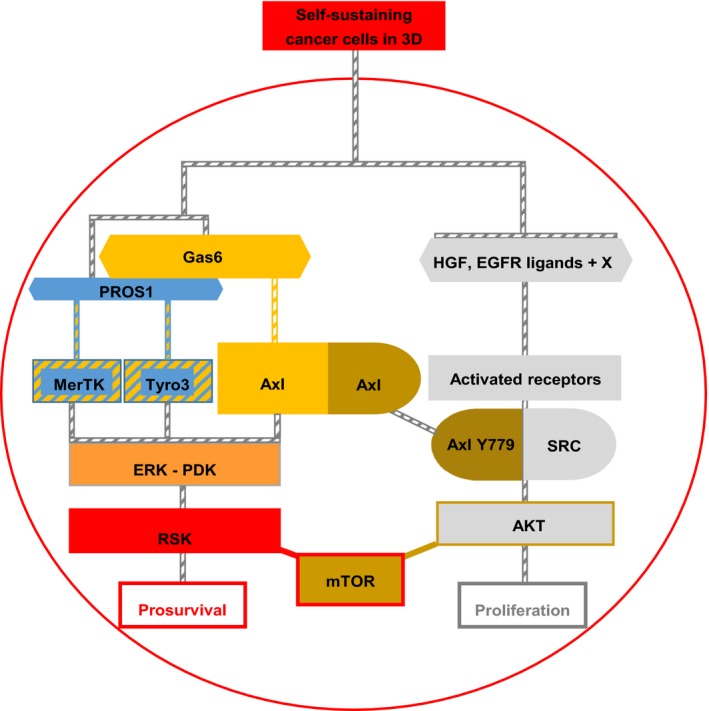
Model of self‐sustaining cancer cells in 3D. Self‐sustaining cancer cells depend on autocrine loops of ligands leading to TAM‐dependent RSK activation and SRC‐dependent AKT pathway activation. In the case of a dominantly activated SRC‐AKT pathway, mTOR signaling might be overdriven. This is partially prevented by inactivation of the TAM ligand system. A double role is ascribed to AXL being involved in both pathways. Availability of TAM‐dependent RSK pathway activation is crucial for survival of cancer cells in case of compound treatment or malnutrition.

### 3.9 Functionality of self‐sustaining cancer cells in 3D

RSK activation by autocrine GAS6 and TAM is crucial for tumor cell survival. RSK pathway activation leads to mTOR phosphorylation and to the phosphorylation of additional RSK‐specific targets. Tumor cells differ in their expression profile of TAM RTKs and PROS1. We postulate that the TAM/PROS1 expression profile might be linked to the expression of other RTK ligands leading to the phosphorylation of SRC. Furthermore, we have proven that the SRC‐dependent transactivation of RTKs and the TAM‐dependent RSK activation display essential prerequisites of self‐sustaining cancer cell proliferation. Therefore, AXL executes a dual function. AXL is transactivated by SRC specifically at tyrosine 779 in GAS6‐independent fashion (Fig. S1), whereas other yet nondefined AXL phosphorylation sites as detected by tyrosine immunoprecipitation are involved in the GAS6‐dependent AXL phosphorylation and subsequent activation of RSK. The treatment of Caliper cells with 0.39 μm BMS777607 leads to an equal activation of both pathways resulting in an efficient resistance reaction based on autocrine GAS6 and HGF secretion and RTK activation. This is different in self‐sustaining cell lines with preference for either RSK activation like MDA‐MB231 or a predominant SRC‐AKT pathway activation like H292. Consequently, just exclusive activation of the RSK or SRC‐AKT pathway leads to insufficient and attenuated resistance reaction to AXL TKIs. We therefore ascribe the role of a limiting factor to AXL in overcoming the imbalance of pathway activation in MDA‐MB231 and H292 cells.

### 3.10 HGF transactivates AXL Y779 in Caliper and MDA‐MB231 cells

Additionally, we examined the impact of antagonistic HGF antibody on phosphorylation of pAXL Y779 in combination with 97 nm BMS777607 treatment of Caliper and MDA‐MB231 spheroids (Fig. S1). We proved that 97 nm BMS777607 leads to increased phosphorylation of pAXL Y779 as a resistance mechanism. The simultaneous application of nm BMS777607 concentrations and αHGF‐specific antibodies attenuated the phosphorylation of pAXL Y779, displaying a partial transactivation of pAXL Y779 by HGF/MET complexes.

## 4 Discussion

In this study, we demonstrate for the first time the dependence of PDK‐RSK‐mTOR activation on autocrine GAS6 and the TAM family members. We prove that challenging conditions by serum deprivation force self‐sustaining tumor cells to switch from AKT/mTOR to a RSK/mTOR signaling cascade. Therefore, we show that tumor cells characterized by inherent high GAS6 expression bear the prerequisite for the switch to the PDK‐RSK‐mTOR pathway (Figs 1 and 2). The advantage of the RSK pathway was demonstrated in 3D long‐term cultures (3D spheroids), whereby tumor cells subdivide into self‐sustaining or dying tumor types. These self‐sustaining cells permit a broad resistance mechanism to AXL TKI treatment by the dual activation of RSK‐mTOR and SRC‐dependent mTOR pathways in 3D spheroids.

Our results indicate that PDK‐RSK‐mTOR pathway is supported by AXL phosphorylation without involvement of the AXL phosphosite Y779 in 2D + 3D culture conditions (Figs 2 and 7). This PDK‐RSK‐mTOR pathway is predominantly activated in MDA‐MB231 cells based on their high autocrine expression of the TAM‐specific ligands GAS6 and PROS1 (Figs 5C and 7). In this case, we have to take into account that different binding affinities of GAS6 and PROS1 to the TAM receptors imply the possibility of diverse TAM interactions as already described by Tsou and coworkers (Tsou *et al*., 2014).

Flexibility in TAM and ligand interactions is based on the individual cellular expression profile of TAMs and their corresponding ligands which guarantee the required efficiency of the RSK‐mTOR pathway activation in coordination with activated AKT‐mTOR in 3D spheroids.

This becomes obvious as H292 cells, lacking MERTK expression, are generally capable of activating RSK (Fig. 1) but on the other side shut down RSK phosphorylation, when AKT‐mTOR becomes predominantly activated in 3D spheroids (Fig. 7).

The interdependence of TAM members in coordination with AKT is proved by the literature. The study of Brown and coworkers shows that TYRO3 and AXL phosphorylations seem to be interdependent in the absence of MERTK. The overexpression experiments with TYRO3 and AXL in Rat2 cells indicate that TYRO3 is therefore responsible for ERK activation (Brown *et al*., 2012). However, TYRO3 seems to activate AKT when stimulated exogenously by GAS6 in the absence of AXL (Demarest *et al*., 2013). An increased expression of GAS6 was associated with increased MERTK/AXL expression in astrocytoma patient samples. The same study indicates that the knockdown of AXL as well as MERTK by siRNA in the astrocytoma line G12 results in decreased AKT and ERK phosphorylations. In parallel, AXL and MERTK knockdown reduced colony formation and resulted in increased chemosensitivity of these cells (Keating *et al*., 2010).

In more detail, we shed light upon the function of GAS6‐dependent AXL phosphorylation, which differs from AXL Y779 phosphorylation in autocrine self‐sustaining cell systems.

Therefore, we observed that AXL Y779 phosphorylation specifically induced AKT signaling, whereas other AXL phosphorylation sites lead to RSK activation (Figs 4A and 2). Further studies should provide detailed information about the responsible specific tyrosine phosphorylation sites as they are still under investigation. We provide evidence that the inhibition of pAXL Y779, pMET Y1349, and pEGFR Y1173 by dasatinib is linking SRC phosphorylation to the activation of specific phosphorylation sites on these RTKs.

In general, we determined that the SRC‐dependent RTK activation and subsequent stimulation of AKT signaling is an assisting pathway of the AXL TKI feedback loop, but in contrast to the RSK activation not the crucial one. The dependency on RSK activation becomes obvious in BMS777607‐treated H292 cells. The BMS777607 treatment increases AKT‐mTOR phosphorylation levels, but still leads to an ATP loss, because RSK phosphorylation drops in parallel as displayed in Fig. 8B.

Insufficient overall AXL phosphorylation might be the limiting factor for RSK stability, thereby redirecting the signaling pathways to a SRC‐dependent AXL Y779 phosphorylation, subsequently leading to AKT‐mTOR activation.

Another important finding of our study is the dichotomous role of AXL Y779 phosphorylation within the resistance mechanism. In MDA‐MB231, we observed a slight induction of AXL Y779 phosphorylation in comparison with Caliper (Fig. 5D). This is in sharp contrast to the higher GAS6 expression and induction level after BMS777607 treatment in MDA‐MB231 (Figs 1B and 5C). Therefore, we speculate that other ligands than GAS6 might transactivate pAXL Y779 in a SRC‐dependent manner. This is underlined by experiments in Caliper and MDA‐MB231 cells where pAXL Y779 becomes transphosphorylated via HGF/MET activation. The hypothesis of HGF‐driven AXL Y779 phosphorylation was proven by simultaneous treatment with 0.097 μm BMS777607 and HGF‐specific antibodies resulting in decreased phosphorylation of AXL Y779 after αHGF antibody application (Fig. S1). The impact of combined BMS777607 and αHGF treatment was validated in proliferation assays using Caliper cells with the result that ATP decreased after αHGF antibody treatment (Fig. 6).

Simultaneously, we proved the decrease in soluble AXL and soluble MET in 3D Caliper supernatants by αHGF antibody treatment in contrast to αGAS6 antibody application (data not shown). In summary, we conclude that HGF in contrast to GAS6 affects the turnover of MET and AXL induced by stress conditions. Additionally, a similar SRC‐dependent mechanism was described by Ruan and coworkers. They showed that activated SRC initially binds to ligand‐activated VEGFR‐2 and that subsequently activated SRC engages AXL via its juxtamembrane domain to trigger AXL autophosphorylation at Y773 in mice (Ruan and Kazlauskas, 2012). H292 cells show a strong proliferation phenotype in 3D based on activated pEGFR Y1173 by long‐term stress condition (Figs 3A,B and 7). Behind this, we assume an autocrine loop of EGFR ligand activation. EGFR activation in H292 cells proceeds in parallel with the SRC‐dependent AXL Y779 phosphorylation. It appears that pAXL Y779 becomes transactivated via SRC by activated EGFR. The assumption is supported by the increase in pSRC Y416 in H292, which is in contrast to MDA‐MB231 cells showing a decrease in AXL Y779 and SRC Y416 phosphorylation (Fig. 7). Based on this, we assume that AXL Y779 phosphorylation is dependent on activated SRC linking AXL activation to other RTKs. This AXL ligand‐independent AXL‐AKT‐mTOR system becomes specifically activated by challenging conditions. Our study provides evidence that activation of mTOR acts as a point of convergence when activated either by AKT or by RSK. In turn, this means that the preferred upstream mTOR activation pathway decides about the intensity of proliferation and migration. We could show that H292 cells exhibiting a predominantly activated EGFR‐AXL Y779‐SRC‐AKT pathway actively repress the phosphorylation of the RSK (Fig. 7). Consequently, this pathway fuels the proliferation potential of this cell line dramatically as shown in Fig. 3A. The activation of mTOR by different RSK phosphorylation levels is reflected *in vivo* by downregulation of RSK activity in metastasis compared to primary lesions of untreated patients with lung cancer. The analysis of Lara *et al*. revealed that RSK‐positive primary tumors correlated with reduced numbers of secondary lesions and decreased RSK expression in metastases (Lara *et al*., 2011). Based on our results, we hypothesize that tumor cells, driven by autocrine GAS6, activate the TAM‐RSK‐dependent survival pathway during the initial steps of tumorigenesis and secondarily switch to a proliferation mode by activation of the MET and/or EGFR‐dependent SRC‐AKT pathway. Insulin receptor substrate‐1 (IRS‐1) is mostly described as adaptor protein for both the insulin (InR) and the insulin‐like growth factor‐I (IGF‐IR) receptors (Pollak, 2012). In H292, the RTK adaptor protein IRS‐1 Y895 is markedly enhanced from day 2 until day 7 in 3D challenge condition without FBS (Fig. 7). Trastuzumab‐resistant MCF7, however, demonstrates that IRS‐1 associates with EGFR and becomes phosphorylated on tyrosine Y896 in EGF‐dependent manner (Knowlden *et al*., 2008). We therefore assume that EGFR influences significantly the IRS‐1 Y895 phosphorylation in H292 cells. This is in accordance with the 2D challenging conditions where a simultaneous increase in pIRS‐1 Y895 and pEGFR Y1173 was observed (data not shown). In contrast to H292, MDA‐MB231 cells depend on pIRS‐1 S612 activation (Fig. 7). After treatment of MDA‐MB231 cells with BMS777607, pIRS‐1 S612 was dramatically induced in 2D but not in 3D conditions (Figs 2 and 5D). This is in diametrical opposition to the AKT S473 phosphorylation. We therefore conclude that a decreased AKT signaling triggers IRS‐1 S612 phosphorylation.

Andreozzi *et al*. observed an increased IRS‐1 S612 phosphorylation after glucosamine treatment as a reaction to a significant impairment in insulin‐stimulated total tyrosine phosphorylation as well as a specific reduction in IRS‐1 Y608 and Y628 phosphorylation, which possess an important role for binding to PI3K p85 subunit (Andreozzi *et al*., 2004). IRS‐1 S612 phosphosite has also been described as competitive binding site between PI3K and SRC and is connected to transformation activity in mammary cancer cells expressing v‐SRC (Sun and Baserga, 2008). Referring to the literature, we hypothesize that IRS‐1 exerts an allocative function between the survival and the proliferation pathway in connection with SRC in the examined self‐sustaining cancer cells.

## 5 Conclusion

Our results indicate that NSCLC and TNBC self‐sustaining tumor cells in 3D spheroids benefit from the activation of PDK‐RSK‐mTOR pathway in the context of high GAS6 secretion. This survival pathway becomes important after treatment of this self‐sustaining tumor cells with AXL/MET inhibitor BMS777607 or multitargeted TKI sunitinib. Therefore, the cells display increased ATP content as well as cell viability when RSK hyperactivation occurs in combination with enhanced SRC‐dependent signaling activity. Additionally, we elucidate a double role of AXL which can be assigned to RSK‐mTOR as well as SRC‐AKT‐mTOR pathway activation. In consequence, our results lead to identification and elucidation of signaling synergy of therapy‐resistant self‐sustaining TNBC and NSCLC cells based on GAS6 TAM‐dependent PDK‐RSK‐mTOR survival pathway and the AXLY779/EGFR/MET‐driven SRC‐mTOR pathway. Consequently, AXL inhibitors should be used in combination with RSK1/2 or mTOR inhibitors to prevent compensatory signaling. This might enhance the efficacy of targeted anti‐AXL therapies. The final decision about using these drug combinations will of course require additional investigation in preclinical and clinical trials.

## Author contributions

CB and RT designed the study concept, performed data analysis, participated in data acquisition, and wrote the manuscript. AU revised the manuscript.

## Supporting information


**Fig. S1** Cross‐activated AXL Y779 phosphorylation by HGF.

**Fig. S2** Western blotting quantification. Relative protein quantification level of Figure 1.

**Fig. S3** Western blotting quantification. Relative protein quantification level of Figure 2 MDA‐MB231 and Caliper cell line.

**Fig. S4** Western blotting quantification. Relative protein quantification level of Figure 2 Hs578T and H292 cell line.

**Fig. S5** Western blotting quantification. Relative protein quantification level of Figure 4.

**Fig. S6** Western blotting quantification. Relative protein quantification level of Figure 5C and 5D.

**Fig. S7** Western blotting quantification. Relative protein quantification level of Figure 5G.

**Fig. S8** Western blotting quantification. Relative protein quantification level of Figure 7 H292 and MDA‐MB231 cell line.

**Fig. S9** Western blotting quantification. Relative protein quantification level of Figure 7 Hs578T cell line.

**Fig. S10** Western blotting quantification. Relative protein quantification level of Figure 8.
