# Supplementary figures and images for: GAS6‐expressing and self‐sustaining cancer cells in 3D spheroids activate the PDK‐RSK‐mTOR pathway for survival and drug resistance
Source: Mol Oncol. 2017 Jul 26;11(10):1430–47. doi: 10.1002/1878-0261.12109 (PMC5623821; doi:10.1002/1878-0261.12109)

Figure S1

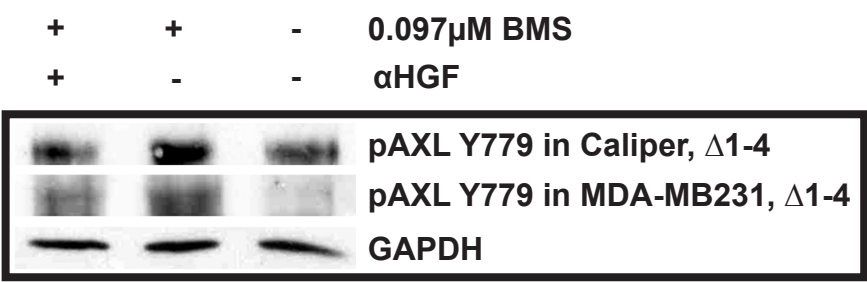

Supplement: Supplementary file 1 — Fig. S1 Cross‐activated AXL Y779 phosphorylation by HGF. [file MOL2-11-1430-s001.pdf]

**Figure S2: Western blotting quantification of Figure 1**

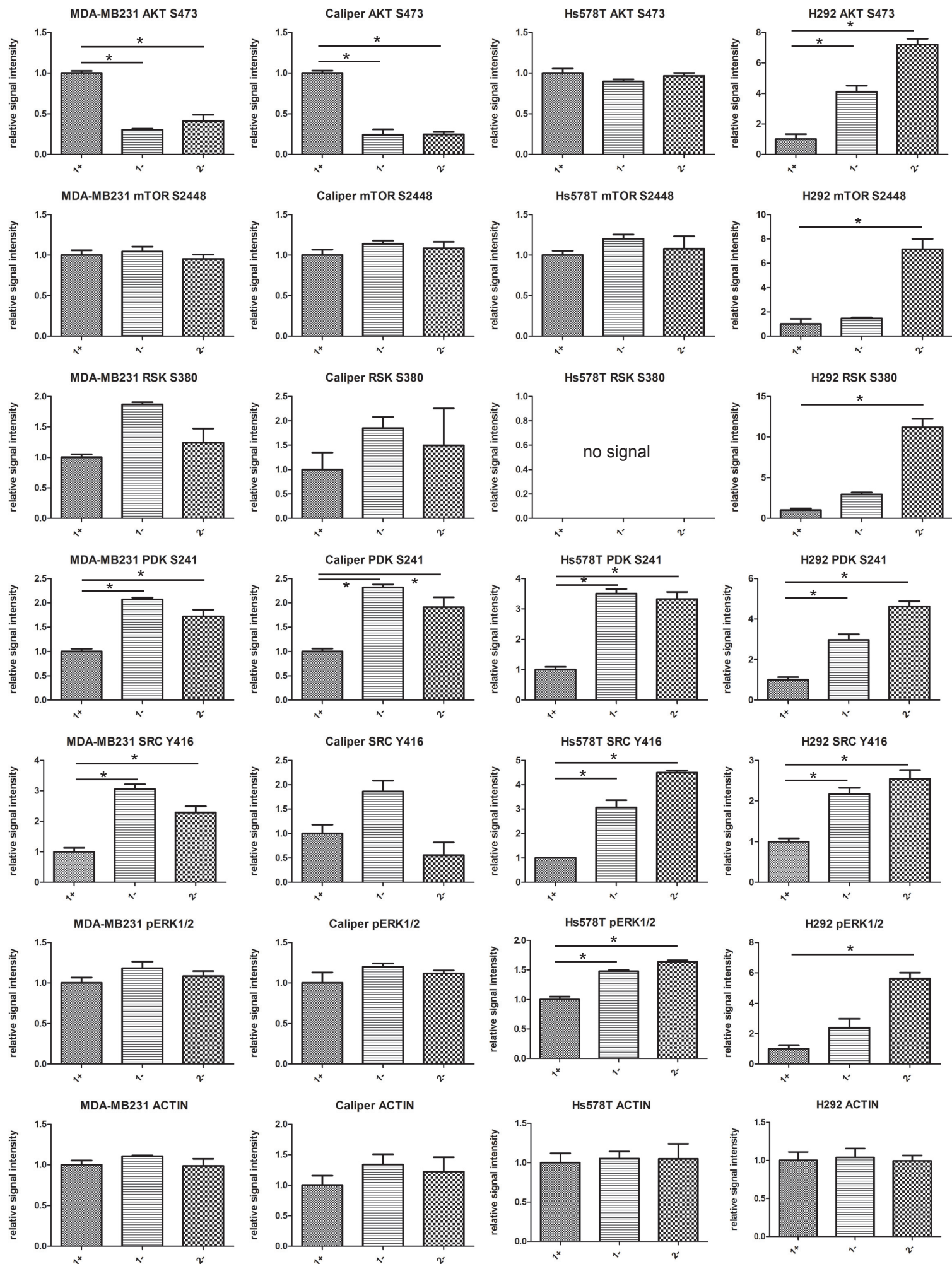

Supplement: Supplementary file 2 — Fig. S2 Western blotting quantification. Relative protein quantification level of Figure 1. [file MOL2-11-1430-s002.pdf]

**Figure S3: Western blotting quantification of Figure 2 MDA-MB231 and Caliper cell line**

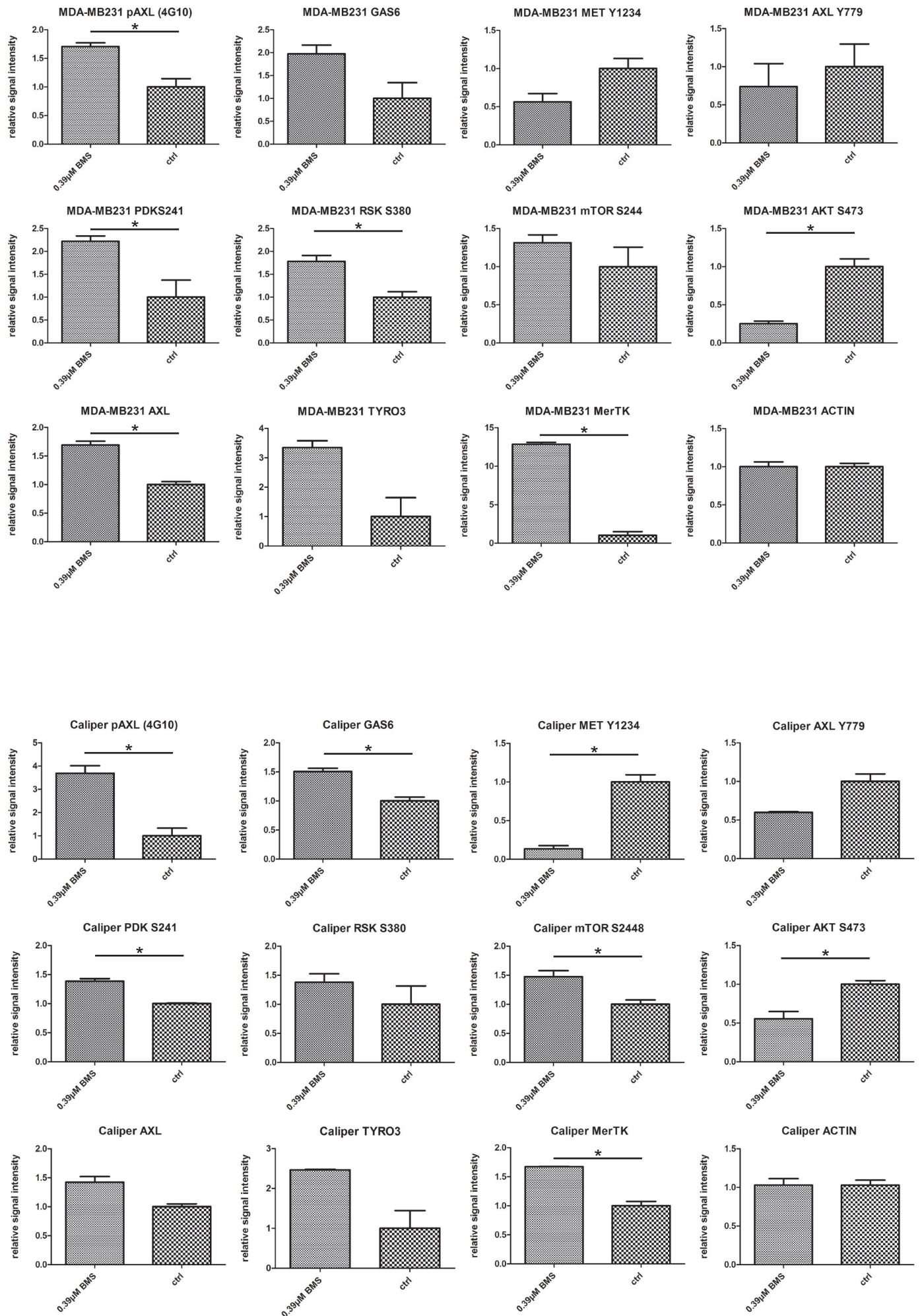

Supplement: Supplementary file 3 — Fig. S3 Western blotting quantification. Relative protein quantification level of Figure 2 MDA‐MB231 and Caliper cell line. [file MOL2-11-1430-s003.pdf]

Figure S4: Western blotting quantification of Figure 2 Hs578T and H292 cell line

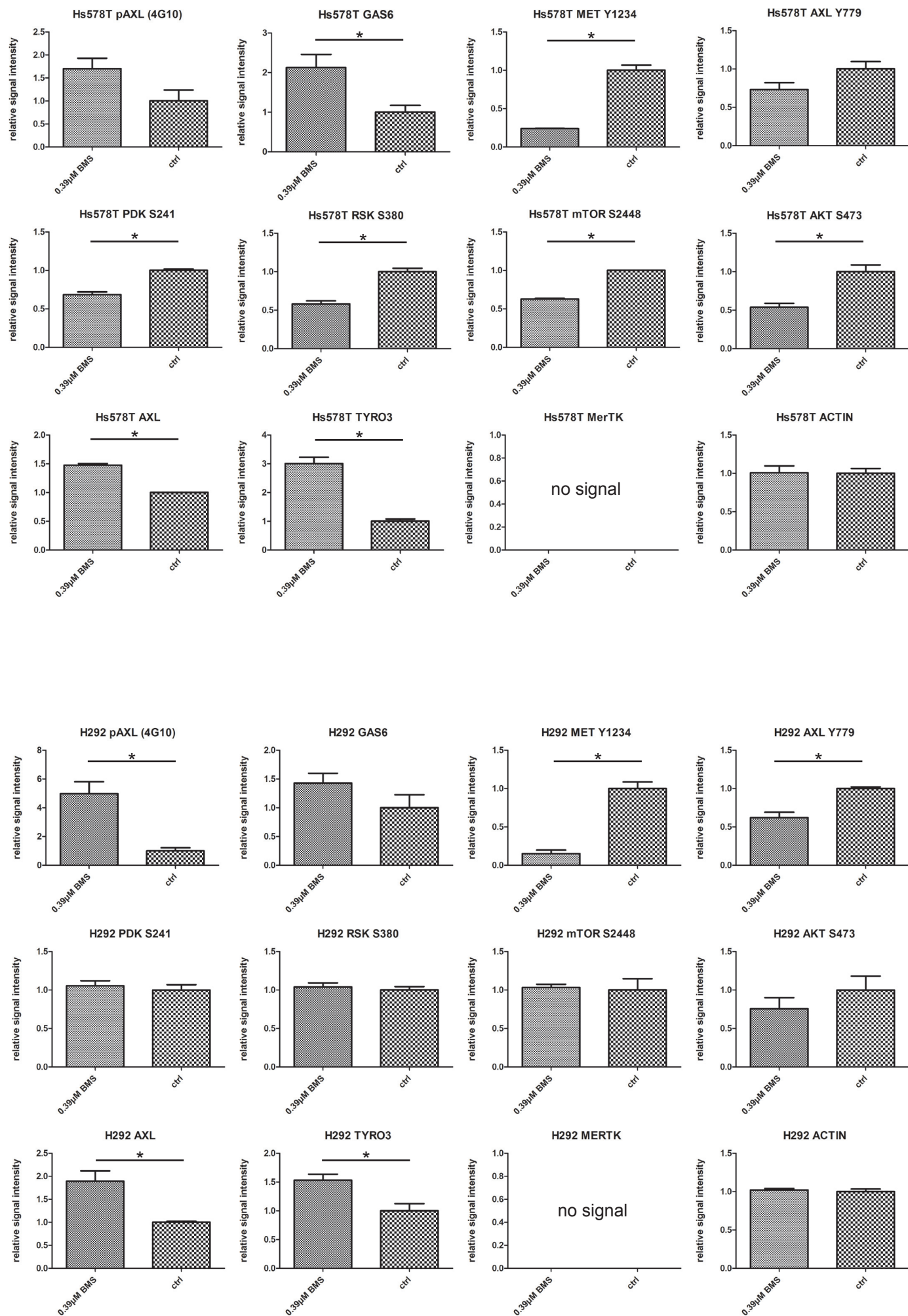

Supplement: Supplementary file 4 — Fig. S4 Western blotting quantification. Relative protein quantification level of Figure 2 Hs578T and H292 cell line. [file MOL2-11-1430-s004.pdf]

**Figure S5: Western blotting quantification of Figure 4**

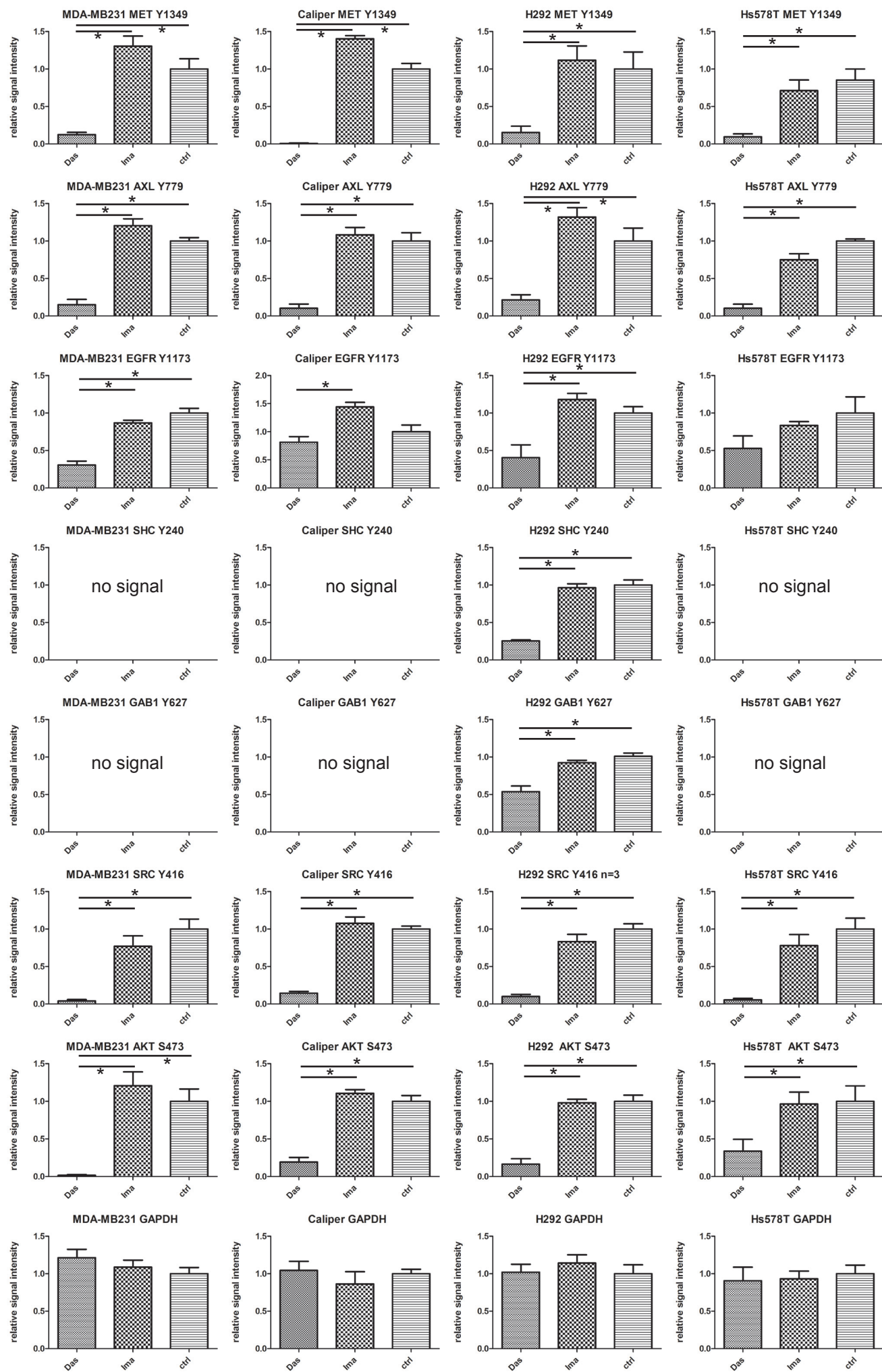

Supplement: Supplementary file 5 — Fig. S5 Western blotting quantification. Relative protein quantification level of Figure 4. [file MOL2-11-1430-s005.pdf]

**Figure S6: Western blotting quantification of Figure 5C and 5D**

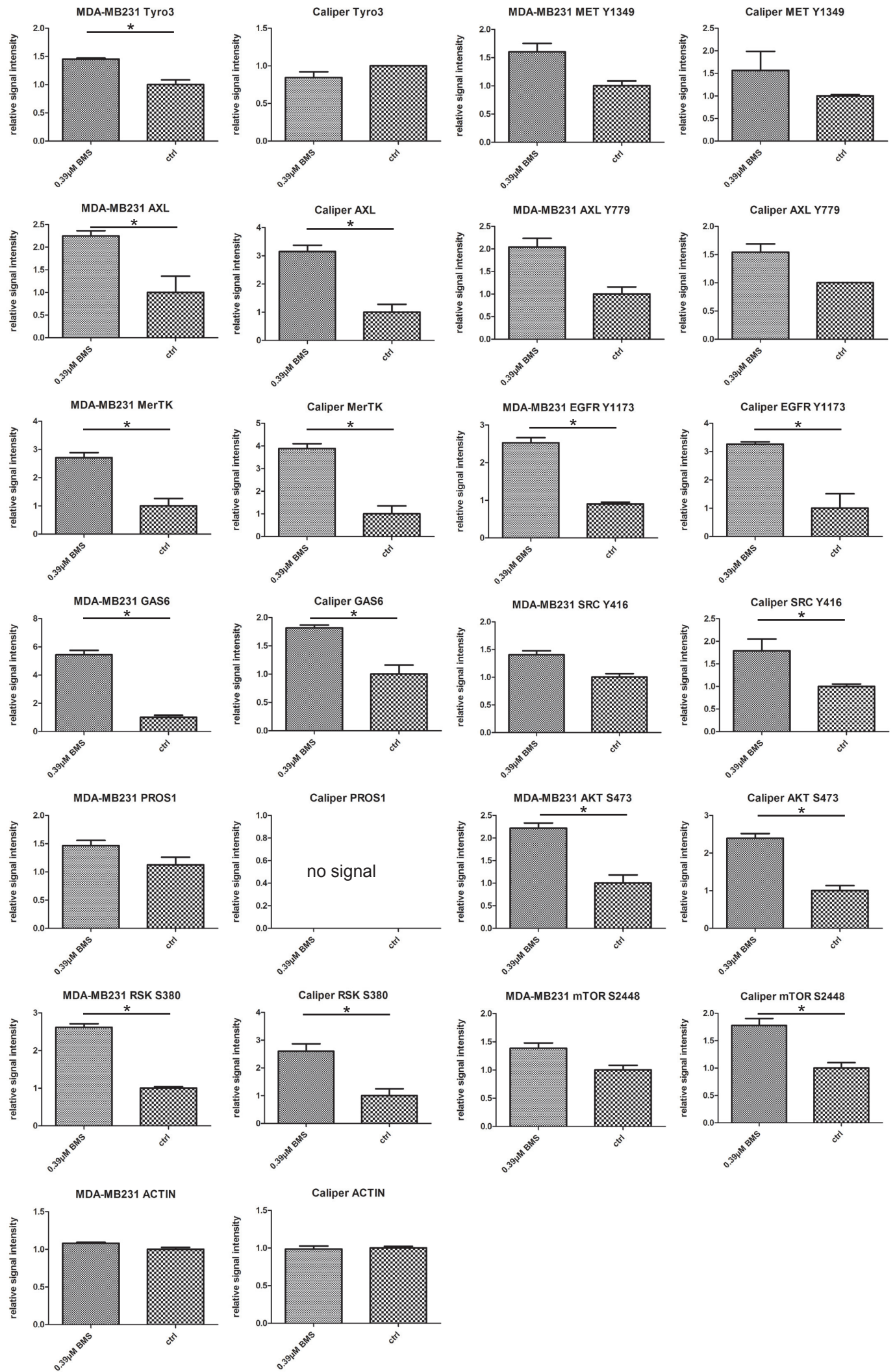

Supplement: Supplementary file 6 — Fig. S6 Western blotting quantification. Relative protein quantification level of Figure 5C and 5D. [file MOL2-11-1430-s006.pdf]

Figure S7: Western blotting quantification of Figure 5G

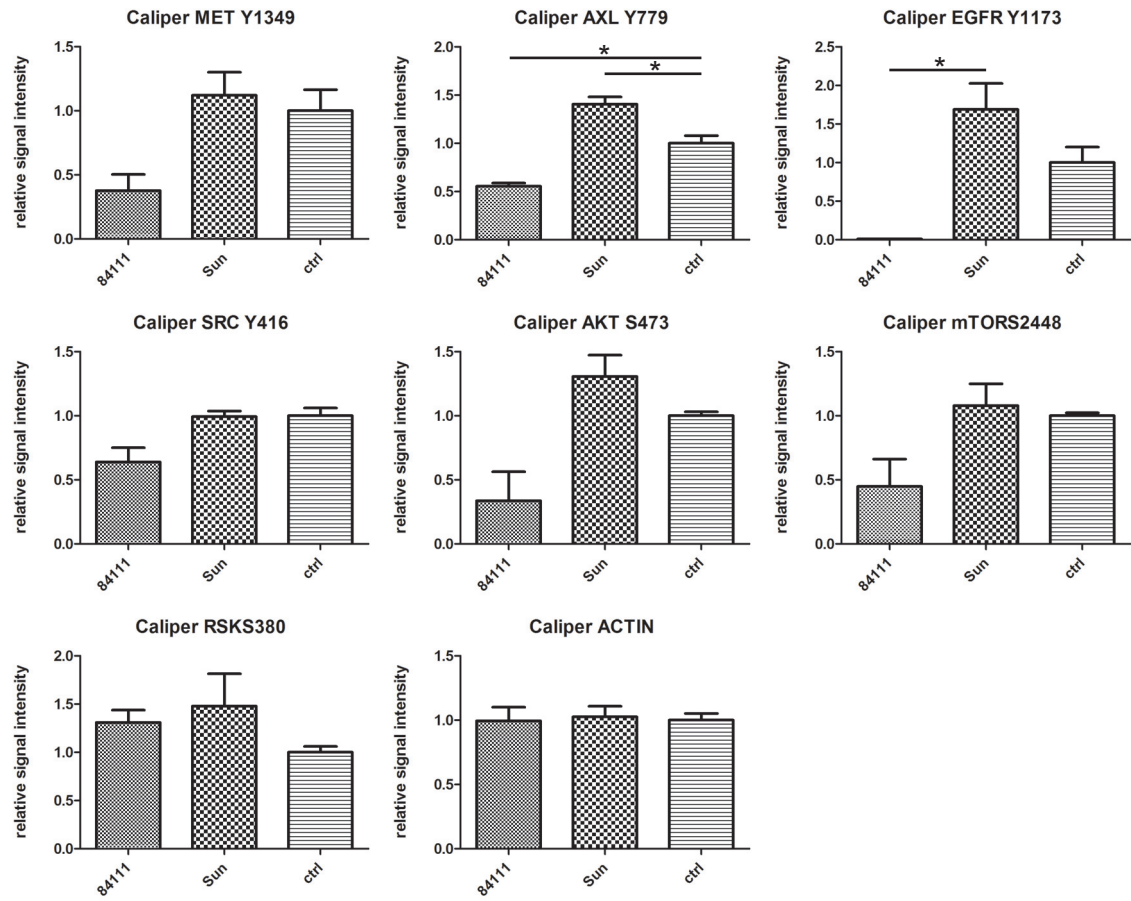

Supplement: Supplementary file 7 — Fig. S7 Western blotting quantification. Relative protein quantification level of Figure 5G. [file MOL2-11-1430-s007.pdf]

**Figure S8: Western blotting quantification of Figure 7 H292 and MDA-MB231 cell line**

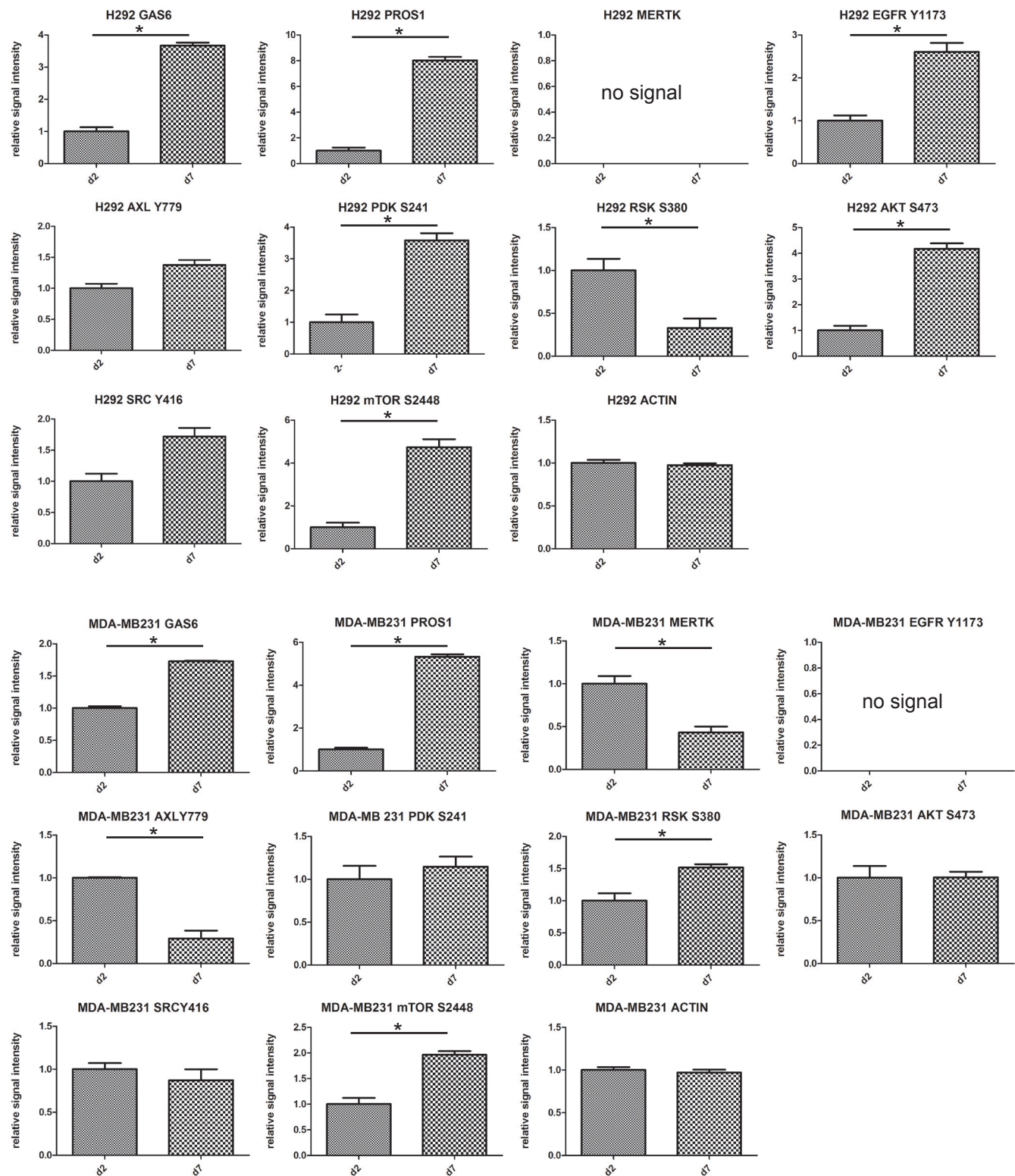

Supplement: Supplementary file 8 — Fig. S8 Western blotting quantification. Relative protein quantification level of Figure 7 H292 and MDA‐MB231 cell line. [file MOL2-11-1430-s008.pdf]

Figure S9: Western blotting quantification of Figure 7 Hs578T cell line

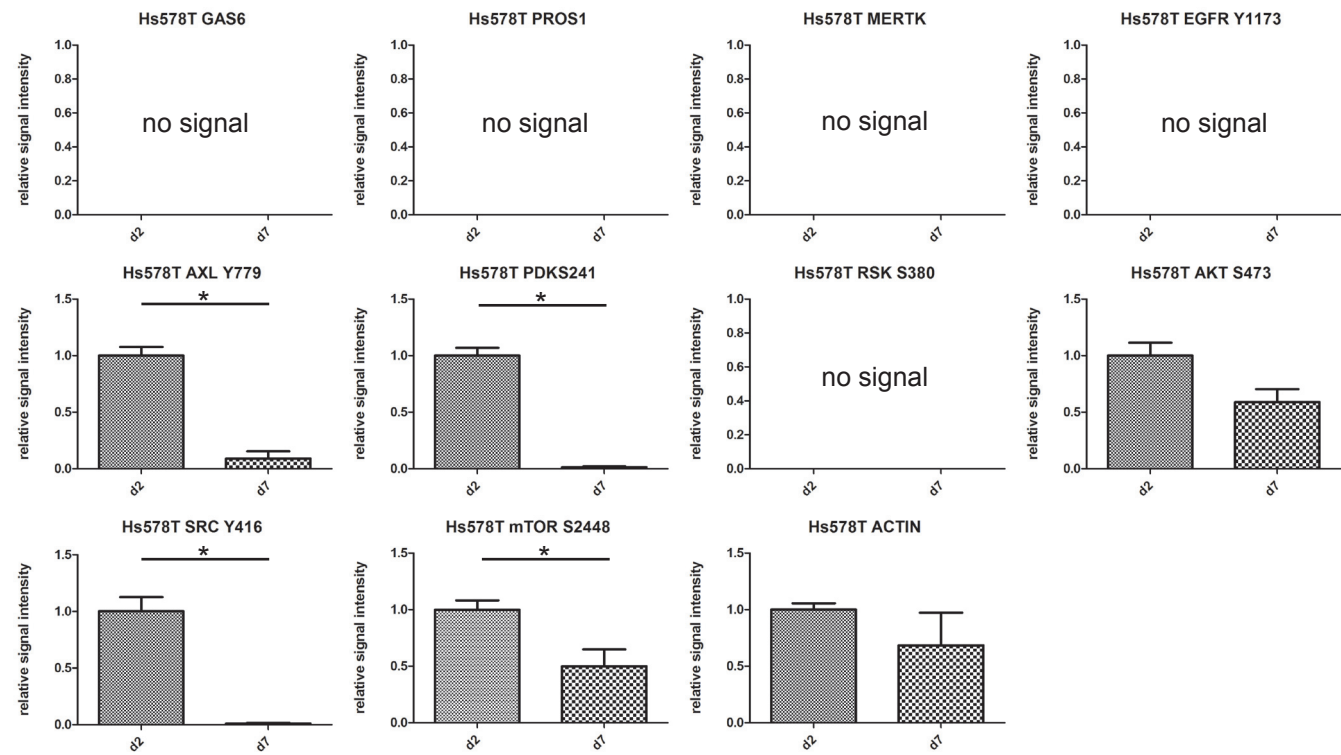

Supplement: Supplementary file 9 — Fig. S9 Western blotting quantification. Relative protein quantification level of Figure 7 Hs578T cell line. [file MOL2-11-1430-s009.pdf]

Figure S10: Western blotting quantification of Figure 8

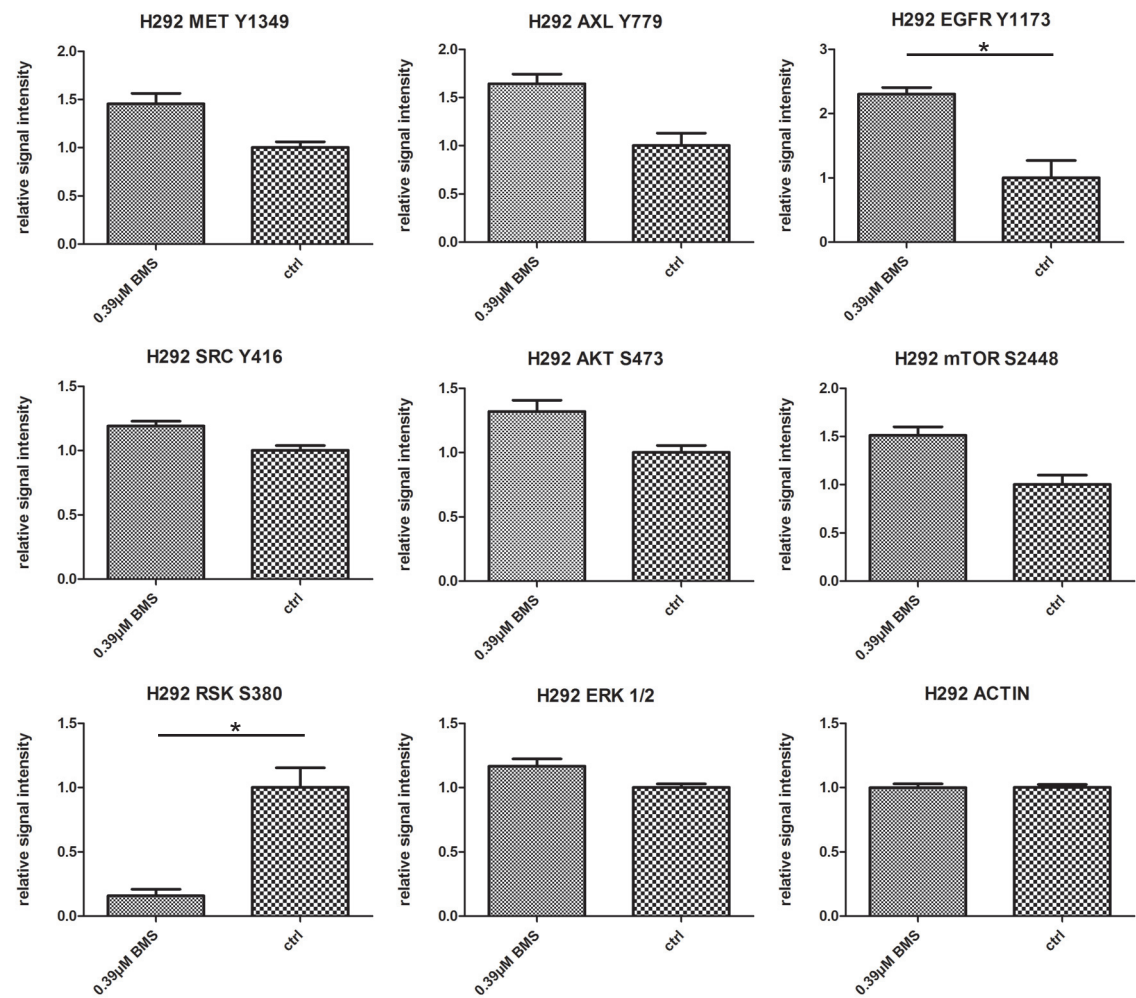

Supplement: Supplementary file 10 — Fig. S10 Western blotting quantification. Relative protein quantification level of Figure 8. [file MOL2-11-1430-s010.pdf]
